# Practical Role of Mutation Analysis for Imatinib Treatment in Patients With Advanced Gastrointestinal Stromal Tumors: A Meta-Analysis

**DOI:** 10.1371/journal.pone.0079275

**Published:** 2013-11-04

**Authors:** Xiaofei Zhi, Xiaoying Zhou, Weizhi Wang, Zekuan Xu

**Affiliations:** 1 Department of General Surgery, the First Affiliated Hospital of Nanjing Medical University, Nanjing, P. R. China; 2 Department of Gastroenterology, the First Affiliated Hospital of Nanjing Medical University, Nanjing, P. R. China; Baylor University Medical Center, United States of America

## Abstract

**Background:**

Imatinib has become the standard first line treatment of gastrointestinal stromal tumors (GIST) in the advanced phase and adjuvant setting. We carried out an up-to-date meta-analysis to determine the practical role of mutation analysis for imatinib treatment in patients with advanced GIST.

**Methods:**

Eligible studies were limited to imatinib treatment for patients with advanced GIST and reported on mutation analysis. Statistical analyses were conducted to calculate the odds ratio (OR), hazard ratio (HR) and 95% confidence interval (CI) using fixed-effects and random-effects models.

**Results:**

A total of 2834 patients from 3 randomized controlled trials and 12 cohort studies were included. The ORs of response rates in KIT exon 11-mutant GISTs were 3.504 (95% CI 2.549-4.816, *p*<0.001) and 3.521 (95% CI 1.731-7.165, *p*=0.001) compared with KIT exon 9-mutant and wild type GISTs, respectively. The HRs of progression-free survival in KIT exon 11-mutant GISTs were 0.365 (95% CI 0.301-0.444, *p*<0.001) and 0.375 (95% CI 0.270-0.519, *p*<0.001) compared with KIT exon 9-mutant and wild type GISTs. The HRs of overall survival in KIT exon 11-mutant GISTs were 0.388 (95% CI 0.293-0.515, *p*<0.001) and 0.400 (95% CI 0.297-0.538, *p*<0.001) compared with KIT exon 9-mutant and wild type GISTs. No statistical significant differences were found between KIT exon 9-mutant and wild type. The overall response rate in KIT-exon 11-mutant GISTs were 70.5% (65%-75.9%) compared with 57.1% (51%-63.2%) in KIT-positive GISTs. No evidence of publication bias was observed.

**Conclusion:**

Patients with advanced GIST harboring a KIT exon 11 mutation have the best response rate and long-term survival with imatinib treatment. Mutation analysis would be more helpful than KIT expression analysis to decide appropriate therapy for a specific patient.

## Introduction

Gastrointestinal stromal tumors (GISTs) are the most common mesenchymal tumors of the gastrointestinal tract [1]. GISTs are thought to derive from intestinal cells of Cajal (ICC) or their precursors and these cells are known to express KIT [2]. KIT (detected as CD117 antigen) encodes a 145 kD receptor kinase and is the homolog of v-KIT, a viral oncogene found in the Hardy-Zuc-kermann 4 feline sarcoma virus. KIT is positive in about 95% of GISTs and has been an important target for diagnosing GIST [3].

The gain-of-function mutations of the tyrosine kinase receptors KIT or platelet-derived growth factor receptor alpha (PDGFRA) have been confirmed to drive the malignant behavior of GISTs [4]. Most frequent mutations in KIT occur in exon 11 (66-71%), followed by exon 9 (13%), exon 13 (1-3%) and exon 17 (1-3%). The remaining GISTs lack mutations in KIT or PDGFRA and therefore termed wild type (WT) (10%) [5].

GIST has become the first solid tumor for tyrosine kinase inhibitor therapy [6]. Imatinib, an inhibitor of ATP-binding domain of certain tyrosine kinase, is the first-line treatment in advanced GISTs and adjuvant setting [7]. Nevertheless, there is no precise strategy to predict which kind of patients can benefit from Imatinib treatment. At present, KIT-positive expression is recommended as indication for Imatinib treatment because all clinical studies of Imatinib treatment for GIST required patients with CD117-positive tumors. Notably, these studies were designed at the time of 2000 to 2001, when it was widely believed that all GISTs were CD117-positive. To date, it is known that 2-5% of GISTs are CD117-negative and many of these harbor a gain-of-function mutation [8]. Moreover, 83-89% of patients with CD117-positive advanced GIST either respond or achieve durable stable disease whereas 11-17% progress [9]. Whether CD117 is suitable to be the indication of Imatinib treatment of GISTs remains further studies. It was reported that patients with GISTs harboring a exon 11 KIT mutation had a better response rate and longer progression-free survival as well as overall survival than either exon 9 KIT mutation or wild type [10]. However, some recent studies obtained a negative result [11,12].

Given all this, in this study, we sought to conduct a meta-analysis to estimate the practical role of mutation analysis for Imatinib treatment in patients with advanced GIST.

## Materials and Methods

### Literature search and selection criteria

Two investigators (XFZ and WZW) performed a systematic literature search in the Cochrane Library, MEDILINE and EMBASE electronic databases, using search terms: “imatinib” and “gastrointestinal stromal tumor”. The two investigators worked independently, at different times and at different medical information centers. Disagreements were resolved through consensus with a third investigator (ZKX). The search was conducted in April 2013 and then repeated in May 2013. Only published studies with full-text articles in English were included. When the same patient population was included in more than one publication, only the most recent or complete study was selected for the meta-analysis.

### Inclusion criteria

Inclusion criteria for primary studies were as follows: (1) patients were required to have a histological diagnosis of CD117-positive or CD117-negative DOG-1-positive GIST, (2) unresectable, metastatic, or recurrent GIST, (3) without any previous Imatinib treatment, (4) Patients could have previously received surgery, but did not undergo any further surgery during Imatinib treatment, (5) Imatinib treatment, (6) mutation analysis (7), assessing the efficacy, progression-free survival or overall survival of Imatinib treatment with different genotypes of GISTs (8), sufficient data for estimating an hazard ratio (HR) with 95% confidence interval

### Quality assessment of primary studies

Quality assessment was performed in each of the acceptable studies in duplicate by independent reviewers (XFZ and WZW) using the Newcastle-Ottawa Quality Assessment Scale for cohort studies and Jadad Scale for RCT studies. Any discrepancies were resolved by a third reviewer (ZKX).

### Data extraction

Two reviewers (XFZ and WZW) independently extracted the required information from all primary studies. Pre-specified data elements included the following: (1) the first author, publication date, study design, sample size, treatment protocol; (2) mutation data including KIT exon 11-mutant cases, KIT exon 9-mutant cases and wild type cases; (3) response data including CR+PR of every genotype (clinical response to Imatinib is classified as complete response (CR), partial response (PR), stable disease (SD), progressive disease (PD), or not assessable (NA) using RECIST criteria); (4) survival data including PFS and OS of every genotype; (5) response data of KIT-positive GIST.

Some studies did not show HR or 95% confidence interval directly for survival data. To be eligible for HR estimation, studies had to report the number of patient with KIT exon 11 mutation, KIT exon 9 mutation and wild type, along with the number of observed cancer progression or death. For studies that included these data, mathematical HR approximation was performed using established methods [13]. In the case that essential data were not given but a Kaplan-Meier curve was available, data were extracted from the survival curve and estimation of HR was performed using the same method. Kaplan-Meier curves were read by Engauge Digitizer version 4.1 (http://digitizer.sourceforge.net).

### Data synthesis

We used the PRISMA checklist as protocol of the meta-analysis and followed the guideline (Table S1). Included studies were divided into four groups for analysis: (1) those with data regarding response rate of different genotypes, (2).those with data regarding PFS, (3).those with data regarding OS, (4). those with data regarding response rate of KIT-positive GIST. Odd ratios (OR) with 95% confidence interval were used to assess the response rate (PR+CR). HRs with 95% confidence interval were used for OS and PFS. Because most clinical studies regarding efficacy of Imatinib only included KIT-positive GISTs, ORs of KIT-positive vs KIT-negative could not be calculated and we combined response rates instead. 

The heterogeneity was initially evaluated by graphical examination of the Forrest plots. Statistical assessment was then performed using a χ^2^-based test. In addition, the I^2^ statistic was calculated to assess the impact of heterogeneity on results. To explore the heterogeneity between studies better, the variables of study design, study quality, year of publication, dose of Imatinib were examined in a meta-regression model. According to the meta-regression results, we further conducted subgroup analyses to explore the possible explanations for the heterogeneity and to examine the impact of different exclusion criteria on the overall result. Meta-analyses were performed using a random-effects model given the inherent between-study heterogeneity (i.e. different sites of GIST, different doses, different study designs, etc.). Sensitivity analyses were carried out to validate whether modification of inclusion criteria affected the results. Potential publication bias was estimated by the funnel plot. All analyses were carried out using Review Manager Version 5 and STATA version 11. Two-sided P < 0.05 was considered statistically significant.

## Results

### Description of studies

**Figure 1:**
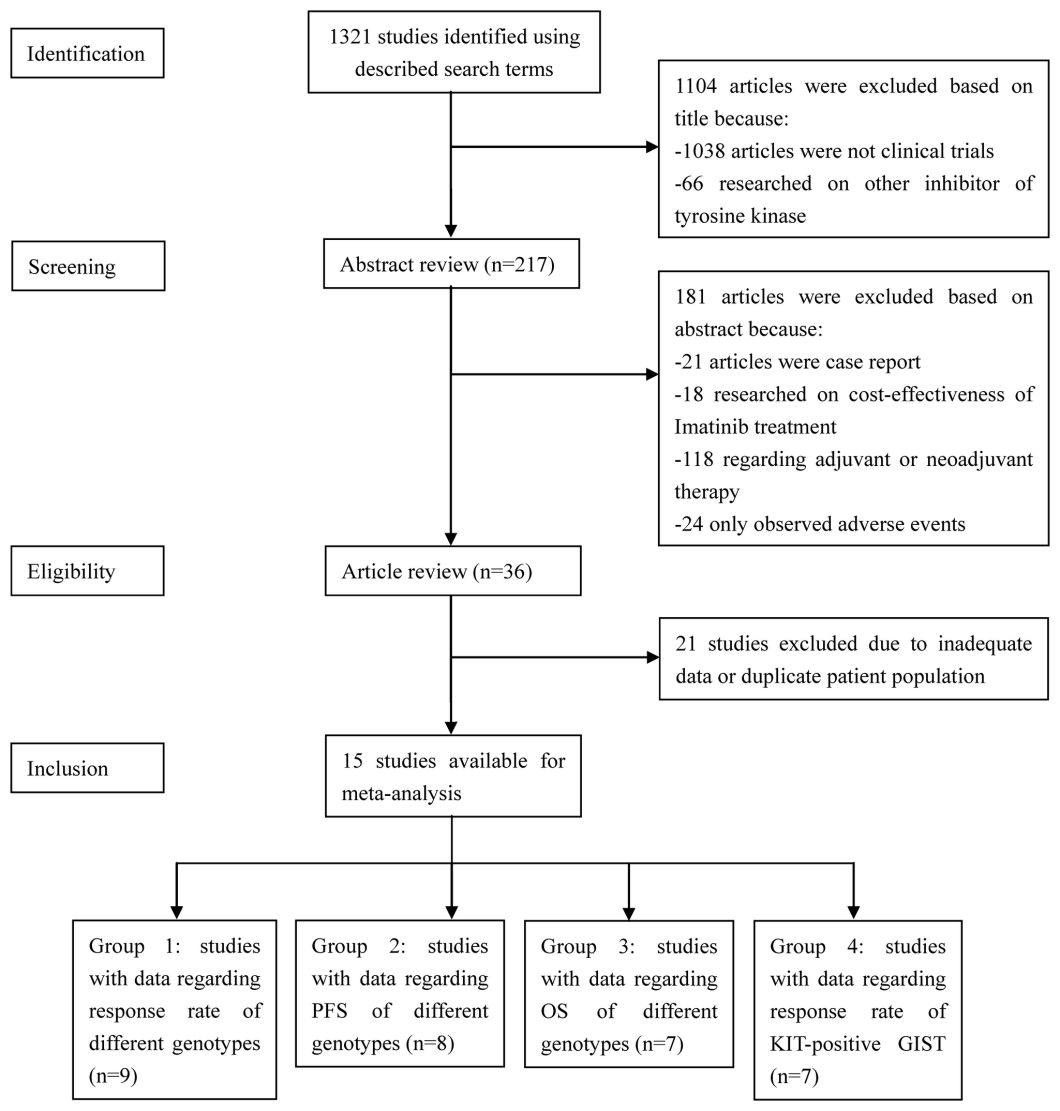
The flow chart of the included studies in the meta-analysis.

Figure 1 is a flow diagram of our search strategy and results. The initial and updated searches together identified 1321 citations. Reviewers identified 36 potential studies with excellent agreement between reviewers. Upon further review, 21 articles were eliminated due to inadequate data for meta-analysis or duplicate patient population. Finally, 15 studies published between 2001 and 2012 met the inclusion criteria [10-12,14-25]. Quality assessment was performed on 12 cohort studies using Newcastle–Ottawa Scale (mean score 6.75 ± 0.45) and 3 RCT studies using Jadad Scale (mean score 4.67 ± 0.58) (Table S2, Table S3).

The characteristics of retained studies are shown in Table 1. In total, 15 studies including 2834 patients were included in the pooled analysis. Of the 15 studies, 9 reported response rate of different genotypes, 8 reported PFS, 7 reported OS and 7 reported response rate of KIT-positive GIST.

**Table 1:** Summary of included studies.

**Studies**	**Design**	**Sample size **	**Dose of Imatinib**
Kang et al, 2012 [22]	retrospective	370	400mg
Gao et al, 2012 [23]	retrospective	158	400mg
Kim et al, 2009 [12]	retrospective	133	400mg
Heinrich et al, 2008 [14]	retrospective	428	400mg or 800mg
Blanke et al, 2008 [15]	prospective	694	400mg or 800mg
Yeh et al, 2007 [24]	prospective	64	400mg
Rutkowski et al, 2007 [25]	prospective	232	400mg
Wardelmann et al, 2006 [11]	retrospective	32	NR
Debiec-Rychter et al, 2006 [16]	retrospective	377	400mg or 800mg
Debiec-Rychter et al, 2004 [19]	retrospective	37	400mg or 600mg or 800mg
Verweij et al, 2004 [17]	prospective	941	400mg or 800mg
Verweij et al, 2003 [20]	prospective	27	400mg or 600mg or 800mg
Heinrich et al, 2003 [10]	prospective	127	400mg or 600mg
Demetri et al, 2002 [18]	prospective	147	400mg or 600mg
van Oosterom et al, 2001 [21]	prospective	36	400mg or 600mg or 800mg

Abbreviations: NR, not reported.

### Estimating HR for PFS and OS

This step was mainly performed according to the procedure described previously. Briefly, 2 HRs ([11,22]) were estimated with the reported data, and the rest ([10,12,14,16,19,23,24],) were calculated with the Kaplan-Meier curve (Table S4).

### Meta-analysis results

The response rates (CR+PR) of different genotypes to Imatinib were tested in 9 reliable studies (group 1 [10,12,14,16,19,22–25]:). Overall meta-analysis showed that the pooled ORs of KIT exon 11-mutant group vs KIT exon 9-mutant group, KIT exon 11-mutant group vs wild type group and KIT exon 9-mutant group vs wild type group were 3.518 (95% CI: 2.556-4.843, p<0.001), 3.521 (95% CI: 1.731-7.165, p=0.001) and 0.981 (95% CI: 0.515-1.868, p=0.953), respectively (Table 2). 

**Table 2:** Pooled OR of response rate.

	**OR (95% CI)**	**z**	***p*-value**	**model**	**test of heterogeneity**		
					**χ^2^**	***p*-value**	**I^2^**
11 vs 9	3.518 (2.556-4.843)	7.71	<0.001	R	4.25	0.834	0.0%
11vs wt	3.521 (1.731-7.165)	3.47	0.001	R	30.08	<0.001	73.4%
9 vs wt	0.981 (0.515-1.868)	0.06	0.953	R	14.72	0.065	45.7%

Abbreviations: OR, odds ratio; CI, confidence interval; F, fixed-effects model; R, random-effects model; 11, KIT exon 11-mutant; 9, KIT exon 9-mutant; wt, wild type.

The HRs of PFS were estimated in 8 studies (group 2 [10–12,14,16,19,22,23]:). Overall meta-analysis showed that pooled HRs of KIT exon 11-mutant group vs KIT exon 9-mutant group, KIT exon 11-mutant group vs wild type group and KIT exon 9-mutant group vs wild type group were 0.365 (95% CI: 0.301-0.444, *p*<0.001), 0.375 (95% CI: 0.270-0.519, *p*<0.001) and 0.905 (95% CI: 0.622-1.316, p=0.601), respectively (Table 3).

**Table 3:** Pooled HR of progression-free survival and overall survival.

	**HR (95% CI)**		**z**	***p*-value**	**model**	**test of heterogeneity**		
						**χ^2^**	***p*-value**	**I^2^**
**PFS**								
11 vs 9	0.365 (0.301-0.444)		10.16	<0.001	R	4.68	0.699	0.0%
11vs wt	0.375 (0.270-0.519)		5.91	<0.001	R	15.28	0.033	54.2%
9 vs wt	0.905 (0.622-1.316)		0.52	0.601	R	12.94	0.074	45.9%
**OS**								
11 vs 9	0.410 (0.271-0.622)		4.19	<0.001	R	9.17	0.164	34.6%
11vs wt	0.400 (0.297-0.538)		6.05	<0.001	R	4.64	0.461	0.0%
9 vs wt	0.887 (0.648-1.216)		0.74	0.457	R	2.99	0.560	0.0%

Abbreviations: HR, hazard ratio; CI, confidence interval; F, fixed-effects model; R, random-effects model; 11, KIT exon 11-mutant; 9, KIT exon 9-mutant; wt, wild type; PFS, progression-free survival; OS, overall survival.

The HRs of OS were estimated in 7 studies (group 3 [10–12,14,16,22,24]:). Overall meta-analysis showed that pooled HRs of KIT exon 11-mutant group vs KIT exon 9-mutant group, KIT exon 11-mutant group vs wild type group and KIT exon 9-mutant group vs wild type group were 0.410 (95% CI: 0.271-0.622, p<0.001), 0.400 (95% CI: 0.297-0.538, p<0.001) and 0.887 (95% CI: 0.648-1.216, p=0.457), respectively (Table 3).

Pooled OR of response rate and HR of PFS/OS showed KIT exon 11-mutant GISTs had the best response and long-term survival to Imatinib treatment. Since most clinical studies of Imatinib treatment for GIST have required patients with CD117-positive tumors, it is not possible to calculated OR of KIT-positive vs KIT-negative for Imatinib treatment. Here, the combined response rate was used to compare different efficacy between KIT exon 11-mutant GIST and KIT-positive GIST. The pooled response rate of KIT exon 11-mutant GIST was calculated from studies of group 1 (Table S5), and KIT-positive GIST from group 4 (Table S6) ([12,15,17,18,20,21,25]). Table 4 indicated that KIT exon 11-mutant GIST had a better response than KIT-positive GIST (70.5% (95% CI: 65%-75.9%) vs 57.1% (95% CI: 51%-63.2%)).

**Table 4:** Pooled response rates for KIT exon 11-mutant GIST and KIT-positive GIST.

	**RR (95% CI)**	**z**	***p*-value**	**model**	**Test of heterogeneity**		
					**χ^2^**	***p***	**I^2^**
exon 11-mutant	70.5% (65%-75.9%)	25.31	<0.001	R	29.33	<0.001	72.7%
KIT-positive	57.1% (51%-63.2%)	18.29	<0.001	R	36.53	<0.001	83.6%

Abbreviations: RR, response rate; CI, confidence interval; R, random-effects model.

All the forest plots are included in Figure S1.

### Meta-regression

Meta-regression analysis indicated that year of publication (P = 0.044) and dose of Imatinib (P = 0.049), but not the study design and study quality, were significant sources of heterogeneity (Table 5).

**Table 5:** Meta-regression analysis.

**Variable**	**Coefficient**	**Standard error**	***p*-value**
**OR: 11 vs wt**			
Study design	-1.013	0.947	0.320
Study quality	1.645	1.332	0.257
Year of publication	-0.280	0.114	**0.044**
Dose of Imatinib	1.635	0.759	**0.049**
**OR: 9 vs wt**			
Study design	-0.668	0.843	0.454
Study quality	1.052	1.234	0.422
Year of publication	-0.176	0.109	0.150
Dose of Imatinib	1.039	0.932	0.170
**HR of PFS: 11 vs wt**			
Study design	0.978	0.626	0.169
Study quality	-0.052	0.629	0.937
Year of publication	-0.002	0.059	0.969
Dose of Imatinib	-0.099	0.369	0.798
**HR of PFS: 9 vs wt**			
Study design	1.341	0.582	0.061
Study quality	-0.122	1.488	0.938
Year of publication	0.020	0.071	0.781
Dose of Imatinib	-0.115	0.468	0.814

Abbreviations: OR, odds ratio; HR, hazard ratio; PFS, progression-free survival; OS, overall survival; 11, KIT exon 11-mutant; 9, KIT exon 9-mutant; wt, wild type; data in bold, significant *P*-value.

### Subgroup analysis

Subgroup analysis was conducted by year of publication and dose of Imatinib. Because dose of 400mg/day was adopted since 2007, we divided studies into two subgroups: before 2007 and after 2007. As shown in Table 6, studies published after 2007 had a better pooled OR of response rate and a better HR of overall survival than those published before 2007.

**Table 6:** Subgroup analysis.

		**Number**	**Pooled (95% CI)**		***p*-value**	**test of heterogeneity**	
						***p*-value**	**I^2^**
**OR**							
11 vs 9: Before 2007		3	4.580 (2.762-7.594)		<0.001	0.549	0.0%
After 2007		6	2.953 (1.955-4.460)		<0.001	0.934	0.0%
11vs wt: Before 2007		3	9.274 (4.116-20.88)		<0.001	0.310	14.5%
After 2007		6	2.202 (1.007-4.816)		0.048	0.005	70.2%
9 vs wt: Before 2007		3	1.930 (0.903-4.124)		0.09	0.375	0.0%
After 2007		6	0.721 (0.341-1.528)		0.394	0.114	43.6%
**HR of PFS**							
11 vs 9: Before 2007		4	0.333 (0.247-0.449)		<0.001	0.671	0.0%
After 2007		4	0.391 (0.303-0.505)		<0.001	0.477	0.0%
11vs wt: Before 2007		4	0.353 (0.213-0.584)		<0.001	0.255	26.1%
After 2007		4	0.386 (0.236-0.632)		<0.001	0.014	71.7%
9 vs wt: Before 2007		4	0.814 (0.343-1.933)		0.640	0.047	39.5%
After 2007		4	0.936 (0.606-1.448)		0.768	0.175	62.2%
**HR of OS**							
11 vs 9: Before 2007		3	0.568 (0.157-2.060)		0.39	0.025	72.8%
After 2007		4	0.391 (0.257-0.593)		<0.001	0.613	0.0%
11vs wt: Before 2007		3	0.240 (0.078-0.744)		0.013	0.183	41.1%
After 2007		4	0.417 (0.282-0.617)		<0.001	0.566	0.0%
9 vs wt: Before 2007		3	1.021 (0.377-2.765)		0.967	0.268	18.5%
After 2007		4	0.776 (0.528-1.141)		0.197	0.833	0.0%

Abbreviations: OR, odds ratio; HR, hazard ratio; PFS, progression-free survival; OS, overall survival; 11, KIT exon 11-mutant; 9, KIT exon 9-mutant; wt, wild type.

### Sensitivity analysis

Sensitivity analyses were performed to explore potential sources of heterogeneity and to examine the influence of various exclusion criteria on the overall result. Exclusion of 1 cohort study that scored 6 did not alter the ORs and HRs substantially (Table S7). Restricting analysis to the studies that were RCT or cohort studies for estimating response rate of KIT-positive GISTs yielded similar results (Table S8). Further exclusion of any single study did not materially alter the overall result.

### Publication bias

The shape of the funnel plots did not indicate any evidence of obvious asymmetry (Figure S2). Then, both of the Begg’s test and the Egger’s test showed the absence of publication bias except for HR of exon 11-mutant vs wild type in OS (Table 7).

**Table 7:** Publication bias for all pooled results.

		***p* -value(Begg’s)**	***p* -value(Egger’s)**
OR	11 vs 9	0.348	0.295
	11 vs wt	0.602	0.821
	9 vs wt	0.754	0.724
HR of PFS	11 vs 9	1.000	0.870
	11 vs wt	0.902	0.728
	9 vs wt	0.266	0.561
HR of OS	11 vs 9	1.000	0.787
	11 vs wt	0.133	0.009
	9 vs wt	0.462	0.388
Response rate	Exon 11-mutant	0.602	0.897
	KIT-positive	0.230	0.115

Abbreviations: OR, odds ratio; HR, hazard ratio; PFS, progression-free survival; OS, overall survival; 11, KIT exon 11-mutant; 9, KIT exon 9-mutant; wt, wild type.

## Discussion

To the best of our knowledge, this is the first meta-analysis to demonstrate a significant benefit from Imatinib treatment for advanced GISTs who harbor a KIT exon 11 mutation in progression-free survival and overall survival, and more importantly, this study may suggest that KIT exon 11 mutation is a more efficient indication for GISTs to receive Imatinib treatment. Compared with KIT exon 9 mutation and wild type, KIT exon 11 mutation delivers a 60% improvement in PFS and OS. It is important to note that some recent studies obtained negative results [11,24]. The pooled results generated in the present study thus conform the long-term benefit of KIT exon 11-mutant GISTs from Imatinib treatment.

This is the second meta-analysis to investigate the cumulative response of different KIT mutations to Imatinib. Previous meta-analysis report similar findings with a 2.29 fold improvement in cumulative response of KIT exon 11-mutant group compared with exon 9-mutant group [26]. Importantly, the previous study did not include the classic EORTC 62005 trial [17] and the newest two trials [22,23] which incorporated KIT-negative GISTs. In fact, KIT-negative GISTs may also harbor a gain-of-function mutation and many of them could benefit from Imatinib [14]. 

Our meta-analysis provides an opportunity to comment on current clinical practice as it relates to the evidence base regarding Imatinib treatment for GIST. The dosage and duration of Imatinib administration had been evaluated for advanced GISTs and adjuvant setting in recent phase III trials [7,15,17]. However, all these large clinical trials were designed at the time of 2000 to 2001 and at that time it was believed that all GISTs were CD117-positive. As a result, in clinical practice, KIT-positive expression is considered as the indication of efficacy of Imatinib. To date, it is known that 2-5% of GISTs are CD117-negative and many of these harbor a gain-of-function mutation [27]. Moreover, data from one of large phase III clinical trials also suggested CD117-negative GISTs might benefit from Imatinib treatment [14]. In addition, even in CD117-positive patients with Imatinib treatment, 83-89% of patients with advanced GIST either respond or achieve durable stable disease whereas 11-17% progress [9]. Therefore, using KIT-positive expression as indication of Imatinib treatment may ignore the benefit of some of KIT-negative GISTs and fail some of KIT-positive GISTs. The present meta-analysis indicated that KIT exon 11-mutant GISTs had a better response than KIT-positive (70.5% (95% CI: 65%-75.9%) vs 57.1% (95% CI: 51%-63.2%)). This is easy to understand because KIT exon 11-mutant GISTs may incorporate some of KIT-negative GISTs who can benefit from Imatinib and abandon some of KIT-positive GISTs who fail in Imatinib treatment. Given all this, to employ KIT exon 11 mutation as indication of Imatinib treatment would be better in clinical practice. We have the following theoretical and practical reasons for it. 

Firstly, like the other inhibitors of tyrosine kinase, Imatinib works by means of targeting some certain constitutive activated sites of tyrosine kinase caused by gain-of-function mutations [28]. So it is necessary to choose a proper constitutive activated site of tyrosine kinase as a target for Imatinib. KIT exon 11 mutation changes the juxtamembrane regions which leads to the ATP-binding domain exposure and consequently constitutive activation of KIT. Imatinib can binds the ATP-binding domain and thus prevents the conformational shift to the active form [9]. Our meta-analysis also showed KIT exon 11-mutant GISTs had the best efficacy of Imatinib. Secondly, GIST is gradually believed to be a disease originating from mutations due to more and more novel mutations discovered. Especially in “wild type”, which is not the truly wild type, some new oncogenic mutations were found to activate downstream of the kinases including NF1 mutation, BRAF mutation, succinate dehydrogenase subunit mutations (SDHA, SDHB, SDHC, SDHD) and RAS-family mutations (HRAS, NRAS, KRAS) [29-31]. Last, but not least, a recent phase II clinical trial showed that with Sunitinib treatment, both PFS and OS were significantly improved in patients with KIT exon 9 mutations (19.4 and 26.9 mo) when compared to exon 11 mutations (5.1 and 12.3 mo) [32]. In a preclinical model, the potency of a PDGFRA-mutant kinase inhibitor was more than 100-fold greater than that of Imatinib for inhibition of the Asp842Val mutation and a clinical trial is ongoing [33]. Patients with BRAF-mutated GIST could benefit from an inhibitor of mutated BRAF [34]. All these data indicated that a specific gain-of-function mutation might need a specific inhibitor of tyrosine kinase and mutation analysis would be more helpful than KIT expression analysis to decide appropriate therapy in clinical practice. 

Despite the size of this meta-analysis, our study has some limitations. First, Data were abstracted from published clinical trial results, and therefore, individual patient information was not available. HRs and CIs for the endpoints included in our analysis were not always reported in individual trials. To minimize the error, HRs and CIs were calculated by three investigators (XFZ, WZW and ZKX). Second, all the trials included in this meta-analysis only incorporated KIT-positive GIST but the recent two trials. Despite the relatively low rate of KIT-negative GIST (2-5%), our results might need minute adjustment with data from further clinical trials. Finally, the doses of Imatinib used in these trials were variable until the results from two phase III trials (S0033 and EORTC 62005) were published. 

In conclusion, this study provides clear evidence of the benefit of Imatinib treatment in the patients with GISTs harboring a KIT exon 11 mutation in terms of response rate and long-term survival. Given that GIST is a kind of disease originating from mutations, decision-making should be individualized case by case taking into account various mutations. As for KIT exon 11-mutant GIST, Imatinib is the most effective inhibitor, while the efficacy of other inhibitors for their own optimum mutations remains further studies.

## Supporting Information

Table S1 **PRISMA checklist.**
(DOC)

Table S2 **Quality assessment with the Newcastle-Ottawa Quality Assessment Scale for cohort studies.**
(DOCX)

Table S3 **Quality assessment with the Jadad Scale for RCT studies.**
(DOCX)

Table S4 **Estimated HRs for PFS and OS.**
(DOCX)

Table S5 **Response rate of different genotypes reported in eligible studies.**
(DOCX)

Table S6 **Response rate of KIT-positive GISTs reported in eligible studies.**
(DOCX)

Table S7 **Sensitivity analysis comparing low and high quality studies.**
(DOCX)

Table S8 **Sensitivity analysis comparing RCT and cohort studies.**
(DOCX)

Figure S1 **Forest plots.**
(PDF)

Figure S2 **Funnel plots.**
(PDF)
