# Supplementary material for: Practical Role of Mutation Analysis for Imatinib Treatment in Patients With Advanced Gastrointestinal Stromal Tumors: A Meta-Analysis
Source: PLoS One. 2013 Nov 4;8(11):e79275. doi: 10.1371/journal.pone.0079275 (PMC3817038; doi:10.1371/journal.pone.0079275)
Supplement: Table S2 — Quality assessment with the Newcastle-Ottawa Quality Assessment Scale for cohort studies. (DOCX) [file pone.0079275.s002.docx]

|  | **Quality Indictors From Newcastle-Ottawa Scale** | | | | | | | | | **Score** |
| --- | --- | --- | --- | --- | --- | --- | --- | --- | --- | --- |
| **Studies** | **1** | **2** | **3** | **4** | **5A** | **5B** | **6** | **7** | **8** |  |
| Cohort | | | | | | | | | |  |
| Kang et al, 2012 | Yes | Yes | Yes | No | Yes | No | Yes | Yes | Yes | 7 |
| Gao et al, 2012 | Yes | Yes | Yes | No | Yes | No | Yes | Yes | Yes | 7 |
| Kim et al, 2009 | No | Yes | Yes | No | Yes | No | Yes | Yes | Yes | 6 |
| Heinrich et al, 2008 | No | Yes | Yes | Yes | Yes | No | Yes | Yes | Yes | 7 |
| Yeh et al, 2007 | No | Yes | Yes | Yes | Yes | No | Yes | Yes | Yes | 7 |
| Rutkowski et al, 2007 | No | Yes | Yes | Yes | Yes | No | Yes | Yes | Yes | 7 |
| Wardelmann et al, 2006 | No | Yes | Yes | Yes | Yes | No | Yes | Yes | Yes | 7 |
| Debiec-Rychter et al, 2006 | No | Yes | Yes | Yes | Yes | No | Yes | Yes | Yes | 7 |
| Debiec-Rychter et al, 2004 | No | Yes | Yes | Yes | Yes | No | Yes | Yes | Yes | 7 |
| Heinrich et al, 2003 | No | Yes | Yes | Yes | Yes | No | Yes | Yes | Yes | 7 |
| Verweij et al, 2003 | No | Yes | Yes | Yes | Yes | No | Yes | No | Yes | 6 |
| van Oosterom et al, 2001 | No | Yes | Yes | Yes | Yes | No | Yes | No | Yes | 6 |
