# Supplementary material for: Practical Role of Mutation Analysis for Imatinib Treatment in Patients With Advanced Gastrointestinal Stromal Tumors: A Meta-Analysis
Source: PLoS One. 2013 Nov 4;8(11):e79275. doi: 10.1371/journal.pone.0079275 (PMC3817038; doi:10.1371/journal.pone.0079275)
Supplement: Table S3 — Quality assessment with the Jadad Scale for RCT studies. (DOCX) [file pone.0079275.s003.docx]

| **Studies** | **Quality Indicators From Jadad Scale** | | | | **Score** |
| --- | --- | --- | --- | --- | --- |
|  | **1** | **2** | **3** | **4** |  |
| RCT | | | | | |
| Blanke et al, 2008 | 2 | 2 | 0 | 1 | 5 |
| Verweij et al, 2004 | 2 | 2 | 0 | 1 | 5 |
| Demetri et al, 2002 | 2 | 2 | 0 | 0 | 4 |
