# Supplementary material for: Practical Role of Mutation Analysis for Imatinib Treatment in Patients With Advanced Gastrointestinal Stromal Tumors: A Meta-Analysis
Source: PLoS One. 2013 Nov 4;8(11):e79275. doi: 10.1371/journal.pone.0079275 (PMC3817038; doi:10.1371/journal.pone.0079275)
Supplement: Table S4 — Estimated HRs for PFS and OS. (DOCX) [file pone.0079275.s004.docx]

| **Studies** | **Comparison** | **HR (95% CI) of PFS** | **HR (95% CI) of OS** |
| --- | --- | --- | --- |
| Kang et al, 2012 | 11 vs 9 | 0.41 (0.25-0.68) | 0.48 (0.19-1.17) |
|  | 11vs wt | 0.46 (0.25-0.83) | 0.26 (0.09-0.72) |
|  | 9 vs wt | 1.11 (0.59-2.1) | 0.55 (0.17-1.82) |
| Gao et al, 2012 | 11 vs 9 | 0.33 (0.19-0.56) | NR |
|  | 11vs wt | 0.22 (0.14-0.35) | NR |
|  | 9 vs wt | 0.57 (0.33-0.99) | NR |
| Kim et al, 2009 | 11 vs 9 | 0.20 (0.07-0.58) | 0.18 (0.04-0.80) |
|  | 11vs wt | 0.39 (0.15-1.01) | 0.27 (0.03-2.53) |
|  | 9 vs wt | 1.01 (0.06-16.46) | 0.77 (0.11-5.46) |
| Heinrich et al, 2008 | 11 vs 9 | 0.45 (0.31-0.66) | 0.43 (0.25-0.72) |
|  | 11vs wt | 0.55 (0.40-0.75) | 0.46 (0.30-0.71) |
|  | 9 vs wt | 1.26 (0.79-2.01) | 0.81 (0.53-1.22) |
| Yeh et al, 2007 | 11 vs 9 | NR | 0.24 (0.06-1.01) |
|  | 11vs wt | NR | NR |
|  | 9 vs wt | NR | NR |
| Wardelmann et al, 2006 | 11 vs 9 | 0.63 (0.22-1.84) | 1.68 (0.55-5.28) |
|  | 11vs wt | 0.85 (0.19-3.90) | 0.094 (0.012-0.748) |
|  | 9 vs wt | 0.95 (0.15-5.91) | SS |
| Debiec-Rychter et al, 2006 | 11 vs 9 | 0.31 (0.22-0.44) | 0.32 (0.21-0.48) |
|  | 11vs wt | 0.36 (0.25-0.51) | 0.43 (0.27-0.70) |
|  | 9 vs wt | 1.25 (0.81-1.95) | 1.24 (0.71-2.17) |
| Debiec-Rychter et al, 2004 | 11 vs 9 | 0.33 (0.08-1.40) | NR |
|  | 11vs wt | 0.50 (0.15-1.70) | NR |
|  | 9 vs wt | 1.50 (0.33-6.80) | NR |
| Heinrich et al, 2003 | 11 vs 9 | 0.34 (0.15-0.78) | 0.27 (0.02-4.18) |
|  | 11vs wt | 0.15 (0.05-0.43) | 0.09 (0.01-1.22) |
|  | 9 vs wt | 0.27 (0.10-0.73) | 0.27 (0.02-3.92) |

Table S2
