# Supplementary material for: Practical Role of Mutation Analysis for Imatinib Treatment in Patients With Advanced Gastrointestinal Stromal Tumors: A Meta-Analysis
Source: PLoS One. 2013 Nov 4;8(11):e79275. doi: 10.1371/journal.pone.0079275 (PMC3817038; doi:10.1371/journal.pone.0079275)
Supplement: Table S5 — Response rate of different genotypes reported in eligible studies. (DOCX) [file pone.0079275.s005.docx]

| **Studies** | **Response Rate (CR+PR) of Different Genotypes** | | |
| --- | --- | --- | --- |
|  | **Exon 11-mutant** | **Exon 9-mutant** | **Wild type** |
| Kang et al, 2012 | 63.64% (112/176) | 36.67% (11/30) | 69.57% (16/23) |
| Gao et al, 2012 | 68.52% (74/108) | 47.83% (11/23) | 36.36% (8/22) |
| Kim et al, 2009 | 68.48% (63/92) | 50% (5/10) | 72.73% (8/11) |
| Heinrich et al, 2008 | 63.60% (180/283) | 37.5% (12/32) | 37.31% (25/67) |
| Yeh et al, 2007 | 57.5% (23/40) | 22.22% (2/9) | 60% (3/5) |
| Rutkowski et al, 2007 | 80.77% (42/52) | 44.44% (4/9) | 26.32% (5/19) |
| Debiec-Rychter et al, 2006 | 67.74% (168/248) | 34.48% (20/58) | 23.08% (12/52) |
| Debiec-Rychter et al, 2004 | 83.33% (20/24) | 25% (1/4) | 33.33% (2/6) |
| Heinrich et al, 2003 | 83.53% (71/85) | 47.83% (11/23) | 11.11% (1/9) |

Table S3
