# Supplementary material for: Practical Role of Mutation Analysis for Imatinib Treatment in Patients With Advanced Gastrointestinal Stromal Tumors: A Meta-Analysis
Source: PLoS One. 2013 Nov 4;8(11):e79275. doi: 10.1371/journal.pone.0079275 (PMC3817038; doi:10.1371/journal.pone.0079275)
Supplement: Table S6 — Response rate of KIT-positive GISTs reported in eligible studies. (DOCX) [file pone.0079275.s006.docx]

| **Studies** | **Response Rate (CR+PR) of KIT-positive GISTs** |
| --- | --- |
| Kim et al, 2009 | 67.26% (76/113) |
| Blanke et al, 2008 | 45.24% (314/694) |
| Rutkowski et al, 2007 | 56.47% (131/232) |
| Verweij et al, 2004 | 52.82% (491/941) |
| Verweij et al, 2003 | 70.37% (19/27) |
| Demetri et al, 2002 | 53.74% (79/147) |
| van Oosterom et al, 2001 | 69.44% (25/36) |

Table S4
