# Supplementary material for: Practical Role of Mutation Analysis for Imatinib Treatment in Patients With Advanced Gastrointestinal Stromal Tumors: A Meta-Analysis
Source: PLoS One. 2013 Nov 4;8(11):e79275. doi: 10.1371/journal.pone.0079275 (PMC3817038; doi:10.1371/journal.pone.0079275)
Supplement: Table S7 — Sensitivity analysis comparing low and high quality studies. (DOCX) [file pone.0079275.s007.docx]

|  | **Pooled (95% CI)** | **z** | | ***p*-value** | **model** | **test of heterogeneity** | | |
| --- | --- | --- | --- | --- | --- | --- | --- | --- |
|  |  |  |  |  |  | **χ^2^** | ***p*-value** | **I^2^** |
| **OR** | | | | | | | | |
| 11 vs 9 | 3.626 (2.608-5.041) | | 7.66 | <0.001 | R | 3.70 | 0.814 | 0.0% |
| 11vs wt | 4.149 (1.998-8.614) | | 3.82 | <0.001 | R | 25.87 | 0.001 | 72.9% |
| 9 vs wt | 1.074 (0.543-2.122) | | 0.20 | 0.838 | R | 13.46 | 0.062 | 48.0% |
| **HR of PFS** | | | | | | | | |
| 11 vs 9 | 0.373 (0.306-0.455) | | 9.78 | <0.001 | R | 3.39 | 0.759 | 0.0% |
| 11vs wt | 0.373 (0.260-0.534) | | 5.39 | <0.001 | R | 15.28 | 0.018 | 60.7% |
| 9 vs wt | 0.898 (0.604-1.335) | | 0.53 | 0.595 | R | 12.94 | 0.044 | 53.6% |
| **HR of OS** | | | | | | | | |
| 11 vs 9 | 0.436 (0.282-0.675) | | 3.72 | <0.001 | R | 8.12 | 0.150 | 38.4% |
| 11vs wt | 0.391 (0.277-0.553) | | 5.32 | <0.001 | R | 4.52 | 0.340 | 11.5% |
| 9 vs wt | 0.891 (0.647-1.226) | | 0.71 | 0.477 | R | 2.97 | 0.397 | 0.0% |
