# Supplementary material for: Practical Role of Mutation Analysis for Imatinib Treatment in Patients With Advanced Gastrointestinal Stromal Tumors: A Meta-Analysis
Source: PLoS One. 2013 Nov 4;8(11):e79275. doi: 10.1371/journal.pone.0079275 (PMC3817038; doi:10.1371/journal.pone.0079275)
Supplement: Table S8 — Sensitivity analysis comparing RCT and cohort studies. (DOCX) [file pone.0079275.s008.docx]

|  | **RR (95% CI)** | **z** | ***p*-value** | **model** | **Test of heterogeneity** | | |
| --- | --- | --- | --- | --- | --- | --- | --- |
|  |  |  |  |  | **χ^2^** | ***p*** | **I^2^** |
| **RCT** | | | | | | | |
| KIT-positive | 50.2% (44.4%-56.1%) | 16.85 | <0.001 | R | 10.17 | 0.006 | 80.3% |
| **Cohort** | | | | | | | |
| KIT-positive | 64% (56.4%-71.6%) | 16.47 | <0.001 | R | 6.15 | 0.105 | 51.2% |

RR: Response rate
