# Supplementary material for: Practical Role of Mutation Analysis for Imatinib Treatment in Patients With Advanced Gastrointestinal Stromal Tumors: A Meta-Analysis
Source: PLoS One. 2013 Nov 4;8(11):e79275. doi: 10.1371/journal.pone.0079275 (PMC3817038; doi:10.1371/journal.pone.0079275)
Supplement: Figure S1 — Forest plots. (PDF) [file pone.0079275.s009.pdf]

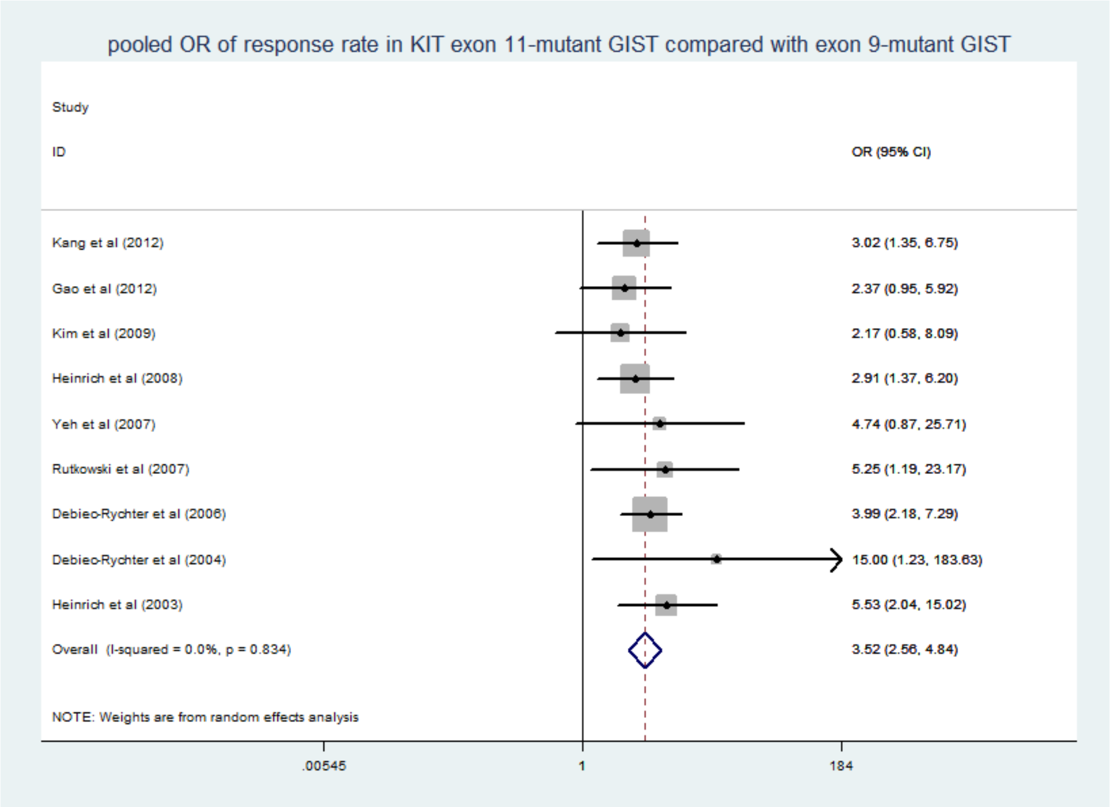
pooled OR of response rate in KIT exon 11-mutant GIST compared with exon 9-mutant GIST
Study
ID
OR (95% CI)
Kang et al (2012)
3.02 (1.35, 6.75)
Gao et al (2012)
2.37 (0.95, 5.92)
Kim et al (2009)
2.17 (0.58, 8.09)
Heinrich et al (2008)
2.91 (1.37, 6.20)
Yeh et al (2007)
4.74 (0.87, 25.71)
Rutkowski et al (2007)
5.25 (1.19, 23.17)
Debiec-Rychter et al (2006)
3.99 (2.18, 7.29)
Debiec-Rychter et al (2004)
15.00 (1.23, 183.63)
Heinrich et al (2003)
5.53 (2.04, 15.02)
Overall (I-squared = 0.0%, p = 0.834)
3.52 (2.56, 4.84)
NOTE: Weights are from random effects analysis
.00545
1
184

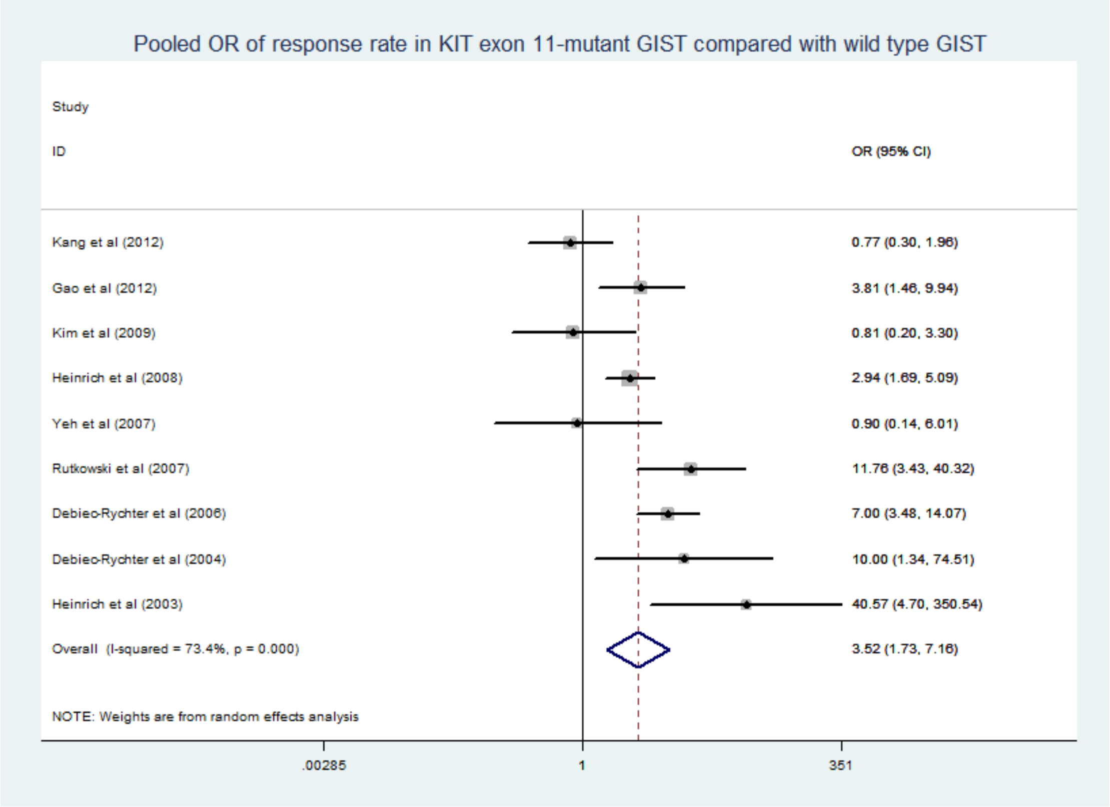
Pooled OR of response rate in KIT exon 11-mutant GIST compared with wild type GIST
Study
ID
OR (95% CI)
Kang et al (2012)
0.77 (0.30, 1.96)
Gao et al (2012)
3.81 (1.46, 9.94)
Kim et al (2009)
0.81 (0.20, 3.30)
Heinrich et al (2008)
2.94 (1.69, 5.09)
Yeh et al (2007)
0.90 (0.14, 6.01)
Rutkowski et al (2007)
11.76 (3.43, 40.32)
Debiec-Rychter et al (2006)
7.00 (3.48, 14.07)
Debiec-Rychter et al (2004)
10.00 (1.34, 74.51)
Heinrich et al (2003)
40.57 (4.70, 350.54)
Overall (I-squared = 73.4%, p = 0.000)
3.52 (1.73, 7.16)
NOTE: Weights are from random effects analysis
.00285
1
351

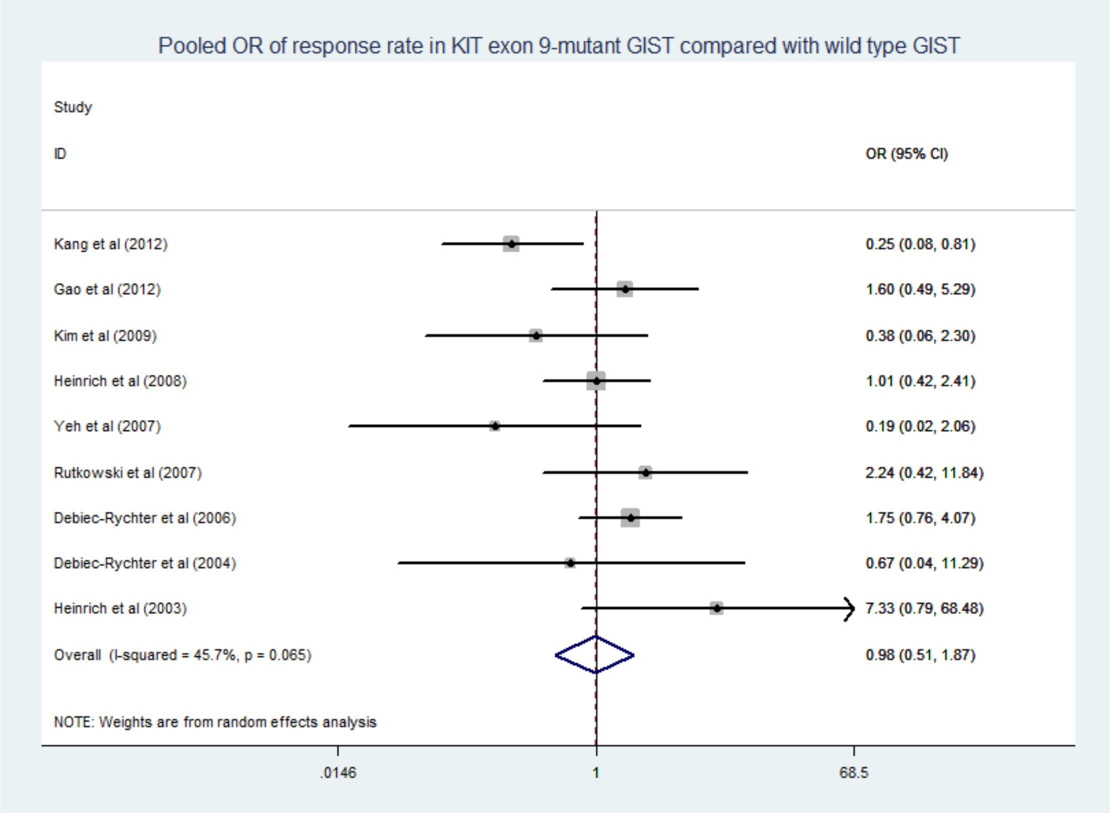
Pooled OR of response rate in KIT exon 9-mutant GIST compared with wild type GIST
Study
ID
OR (95% CI)
Kang et al (2012)
0.25 (0.08, 0.81)
Gao et al (2012)
1.60 (0.49, 5.29)
Kim et al (2009)
0.38 (0.06, 2.30)
Heinrich et al (2008)
1.01 (0.42, 2.41)
Yeh et al (2007)
0.19 (0.02, 2.06)
Rutkowski et al (2007)
2.24 (0.42, 11.84)
Debiec-Rychter et al (2006)
1.75 (0.76, 4.07)
Debiec-Rychter et al (2004)
0.67 (0.04, 11.29)
Heinrich et al (2003)
7.33 (0.79, 68.48)
Overall (I-squared = 45.7%, p = 0.065)
0.98 (0.51, 1.87)
NOTE: Weights are from random effects analysis
.0146
1
68.5

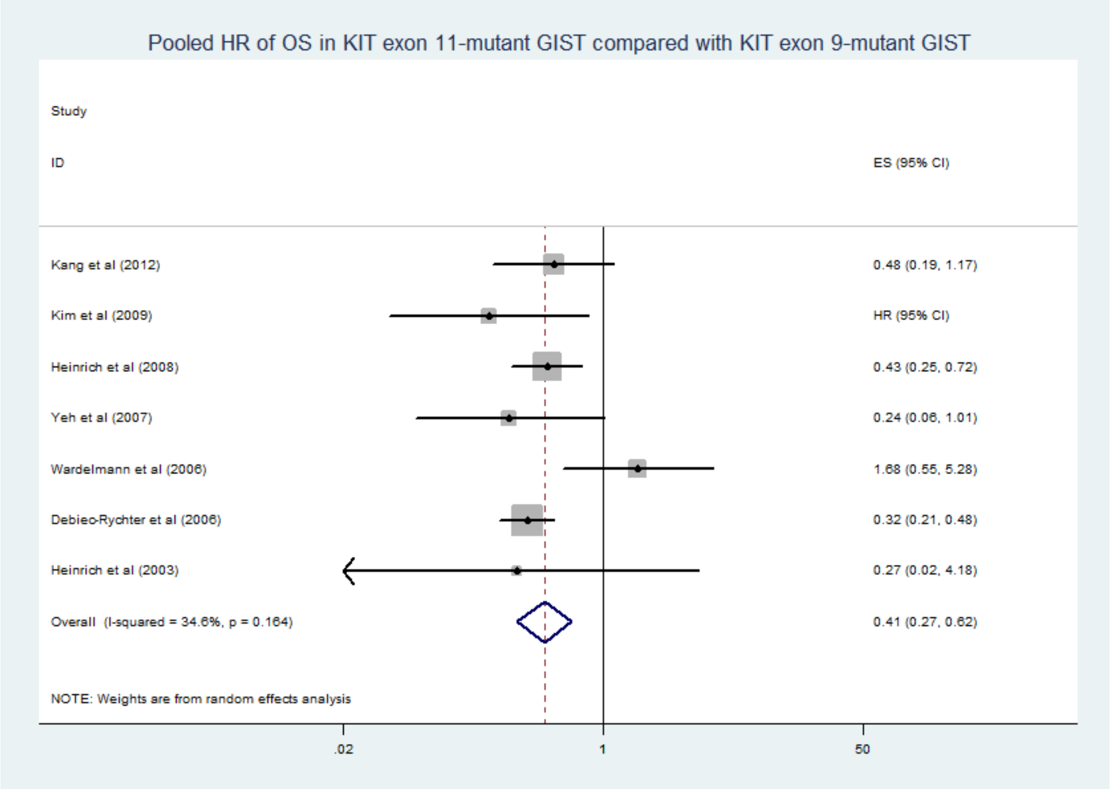
Pooled HR of OS in KIT exon 11-mutant GIST compared with KIT exon 9-mutant GIST
Study
ID
ES (95% CI)
Kang et al (2012)
0.48 (0.19, 1.17)
Kim et al (2009)
HR (95% CI)
Heinrich et al (2008)
0.43 (0.25, 0.72)
Yeh et al (2007)
0.24 (0.06, 1.01)
Wardelmann et al (2006)
1.68 (0.55, 5.28)
Debieo-Rychter et al (2006)
0.32 (0.21, 0.48)
Heinrich et al (2003)
0.27 (0.02, 4.18)
Overall (I-squared = 34.6%, p = 0.164)
0.41 (0.27, 0.62)
NOTE: Weights are from random effects analysis
.02
1
50

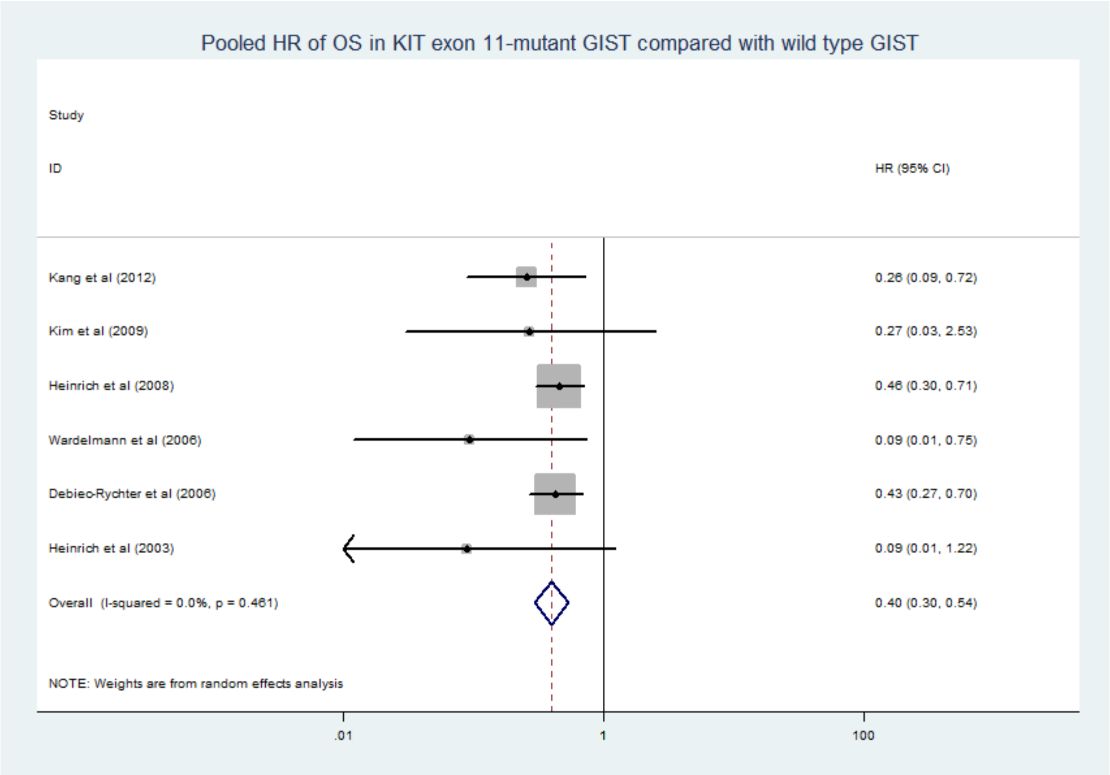
Pooled HR of OS in KIT exon 11-mutant GIST compared with wild type GIST
Study
ID
HR (95% CI)
Kang et al (2012)
0.26 (0.09, 0.72)
Kim et al (2009)
0.27 (0.03, 2.53)
Heinrich et al (2008)
0.46 (0.30, 0.71)
Wardelmann et al (2006)
0.09 (0.01, 0.75)
Debiec-Rychter et al (2006)
0.43 (0.27, 0.70)
Heinrich et al (2003)
0.09 (0.01, 1.22)
Overall (I-squared = 0.0%, p = 0.461)
0.40 (0.30, 0.54)
NOTE: Weights are from random effects analysis
.01
1
100

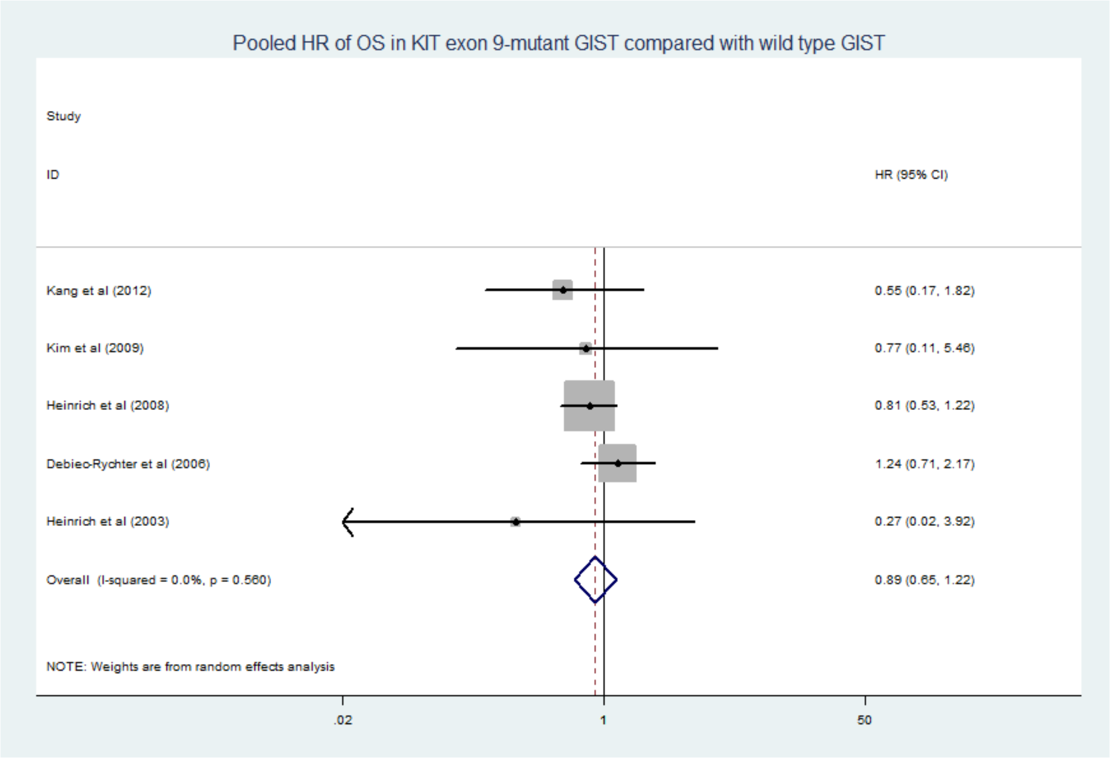
Pooled HR of OS in KIT exon 9-mutant GIST compared with wild type GIST
Study
ID
HR (95% CI)
Kang et al (2012)
0.55 (0.17, 1.82)
Kim et al (2009)
0.77 (0.11, 5.46)
Heinrich et al (2008)
0.81 (0.53, 1.22)
Debiec-Rychter et al (2006)
1.24 (0.71, 2.17)
Heinrich et al (2003)
0.27 (0.02, 3.92)
Overall (I-squared = 0.0%, p = 0.560)
0.89 (0.65, 1.22)
NOTE: Weights are from random effects analysis
.02
1
50

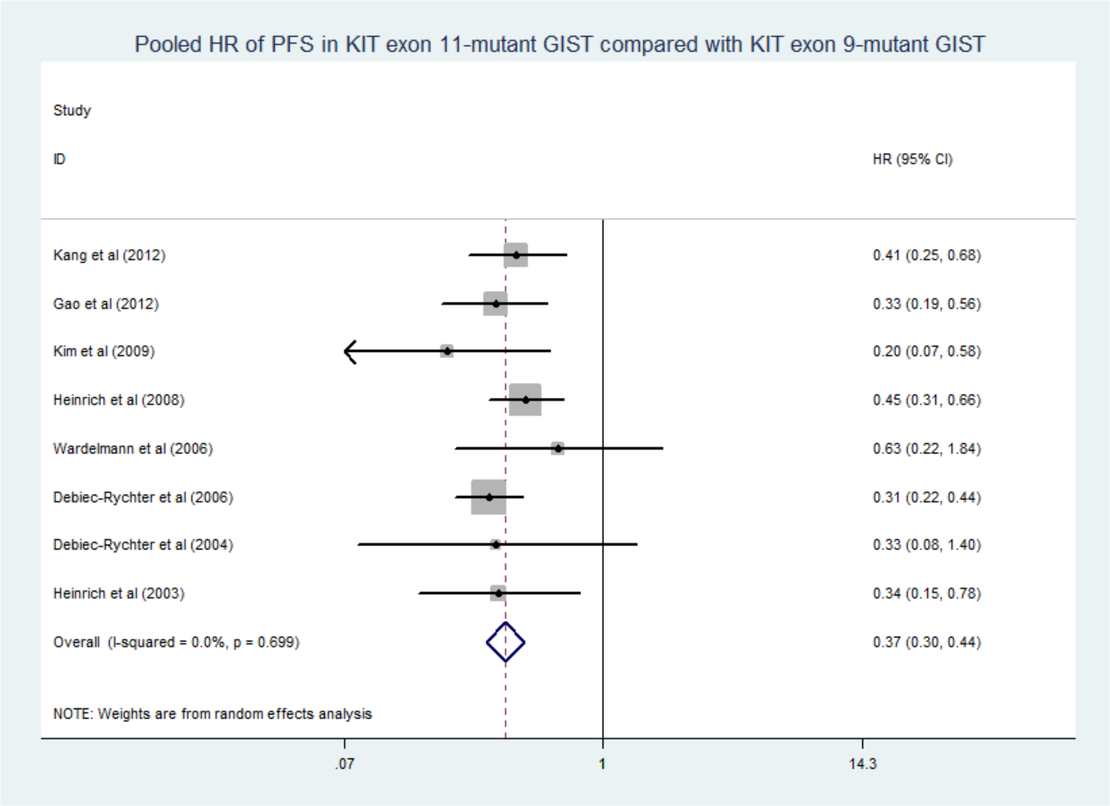

Pooled HR of PFS in KIT exon 11-mutant GIST compared with KIT exon 9-mutant GIST
Study
ID
HR (95% CI)
Kang et al (2012)
0.41 (0.25, 0.68)
Gao et al (2012)
0.33 (0.19, 0.56)
Kim et al (2009)
0.20 (0.07, 0.58)
Heinrich et al (2008)
0.45 (0.31, 0.66)
Wardelmann et al (2006)
0.63 (0.22, 1.84)
Debiec-Rychter et al (2006)
0.31 (0.22, 0.44)
Debiec-Rychter et al (2004)
0.33 (0.08, 1.40)
Heinrich et al (2003)
0.34 (0.15, 0.78)
Overall (I-squared = 0.0%, p = 0.699)
0.37 (0.30, 0.44)
NOTE: Weights are from random effects analysis
.07
1
14.3

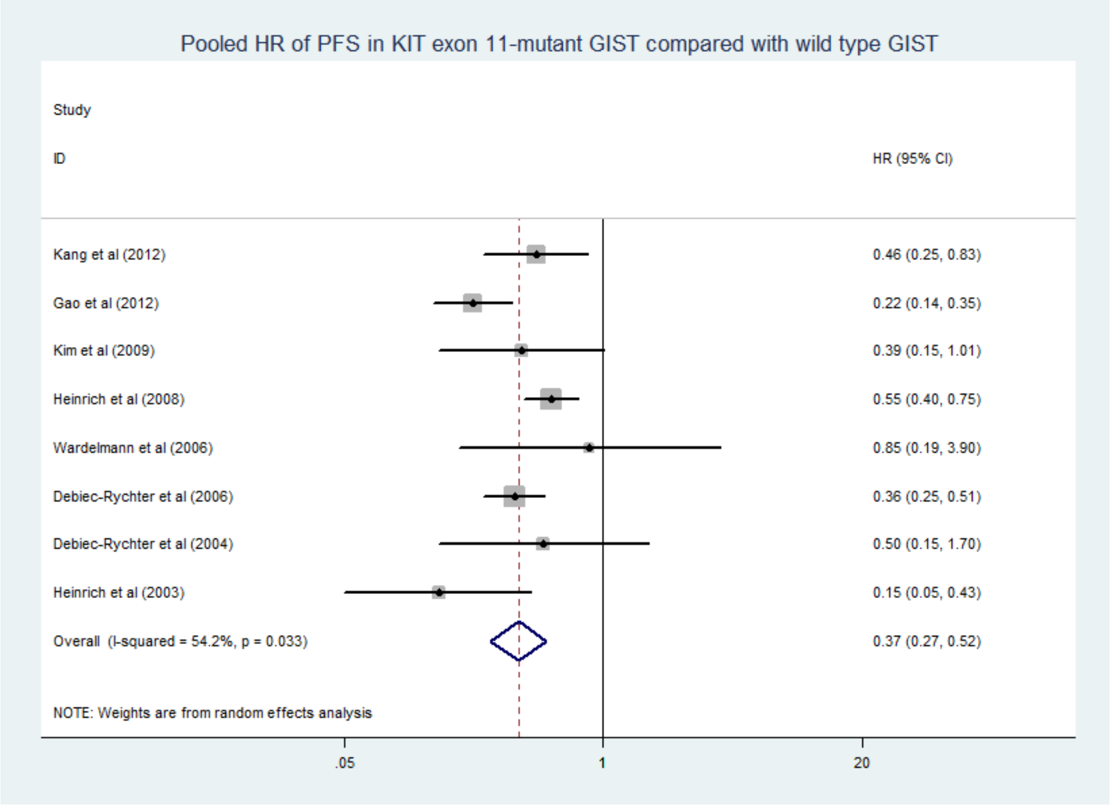

Pooled HR of PFS in KIT exon 11-mutant GIST compared with wild type GIST
Study
ID
HR (95% CI)
Kang et al (2012)
0.46 (0.25, 0.83)
Gao et al (2012)
0.22 (0.14, 0.35)
Kim et al (2009)
0.39 (0.15, 1.01)
Heinrich et al (2008)
0.55 (0.40, 0.75)
Wardelmann et al (2006)
0.85 (0.19, 3.90)
Debiec-Rychter et al (2006)
0.36 (0.25, 0.51)
Debiec-Rychter et al (2004)
0.50 (0.15, 1.70)
Heinrich et al (2003)
0.15 (0.05, 0.43)
Overall (I-squared = 54.2%, p = 0.033)
0.37 (0.27, 0.52)
NOTE: Weights are from random effects analysis
.05
1
20

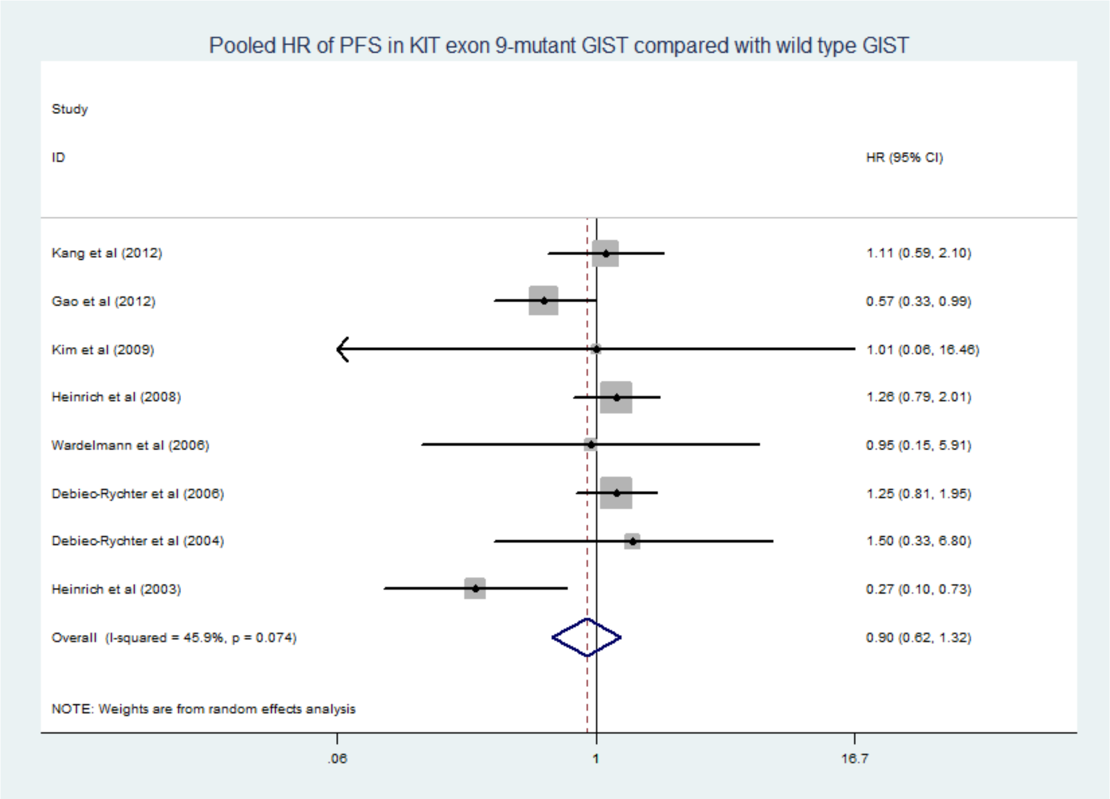
Pooled HR of PFS in KIT exon 9-mutant GIST compared with wild type GIST
Study
ID
HR (95% CI)
Kang et al (2012)
1.11 (0.59, 2.10)
Gao et al (2012)
0.57 (0.33, 0.99)
Kim et al (2009)
1.01 (0.06, 16.46)
Heinrich et al (2008)
1.26 (0.79, 2.01)
Wardelmann et al (2006)
0.95 (0.15, 5.91)
Debiec-Rychter et al (2006)
1.25 (0.81, 1.95)
Debiec-Rychter et al (2004)
1.50 (0.33, 6.80)
Heinrich et al (2003)
0.27 (0.10, 0.73)
Overall (I-squared = 45.9%, p = 0.074)
0.90 (0.62, 1.32)
NOTE: Weights are from random effects analysis
.06
1
16.7

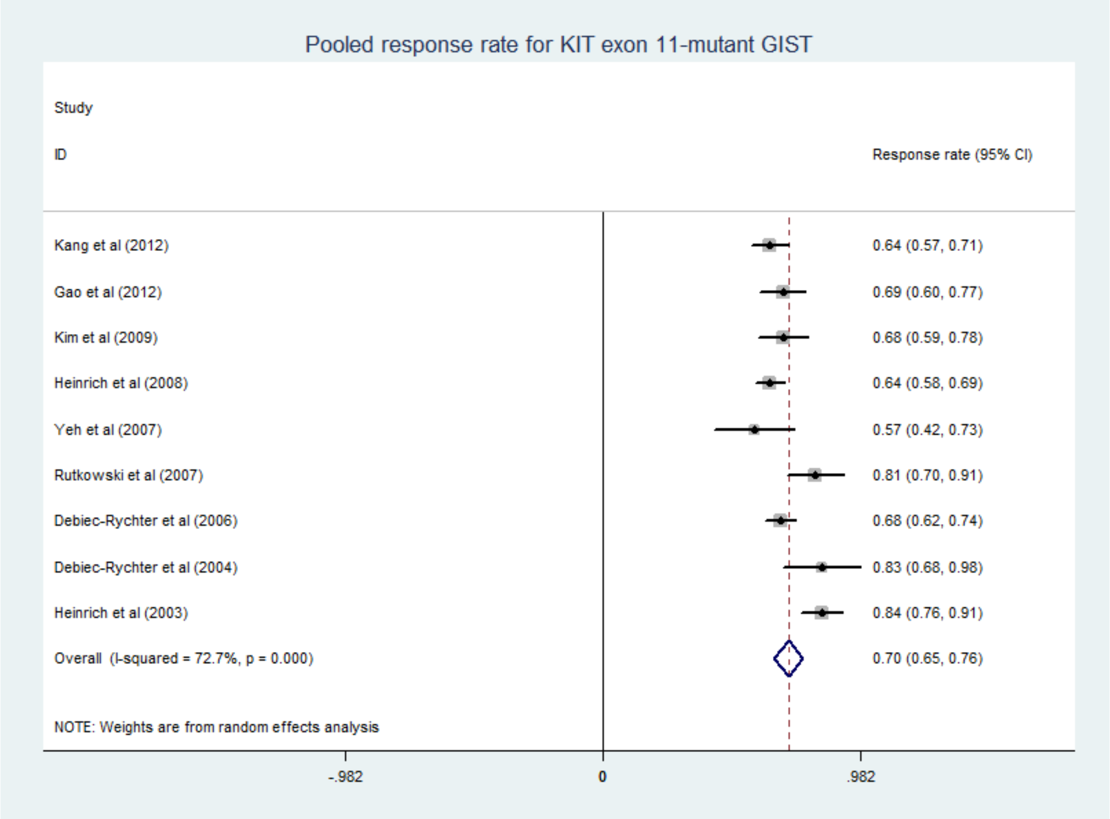
Pooled response rate for KIT exon 11-mutant GIST
Study
ID
Response rate (95% CI)
Kang et al (2012)
0.64 (0.57, 0.71)
Gao et al (2012)
0.69 (0.60, 0.77)
Kim et al (2009)
0.68 (0.59, 0.78)
Heinrich et al (2008)
0.64 (0.58, 0.69)
Yeh et al (2007)
0.57 (0.42, 0.73)
Rutkowski et al (2007)
0.81 (0.70, 0.91)
Debiec-Rychter et al (2006)
0.68 (0.62, 0.74)
Debiec-Rychter et al (2004)
0.83 (0.68, 0.98)
Heinrich et al (2003)
0.84 (0.76, 0.91)
Overall (I-squared = 72.7%, p = 0.000)
0.70 (0.65, 0.76)
NOTE: Weights are from random effects analysis
-.982
0
.982

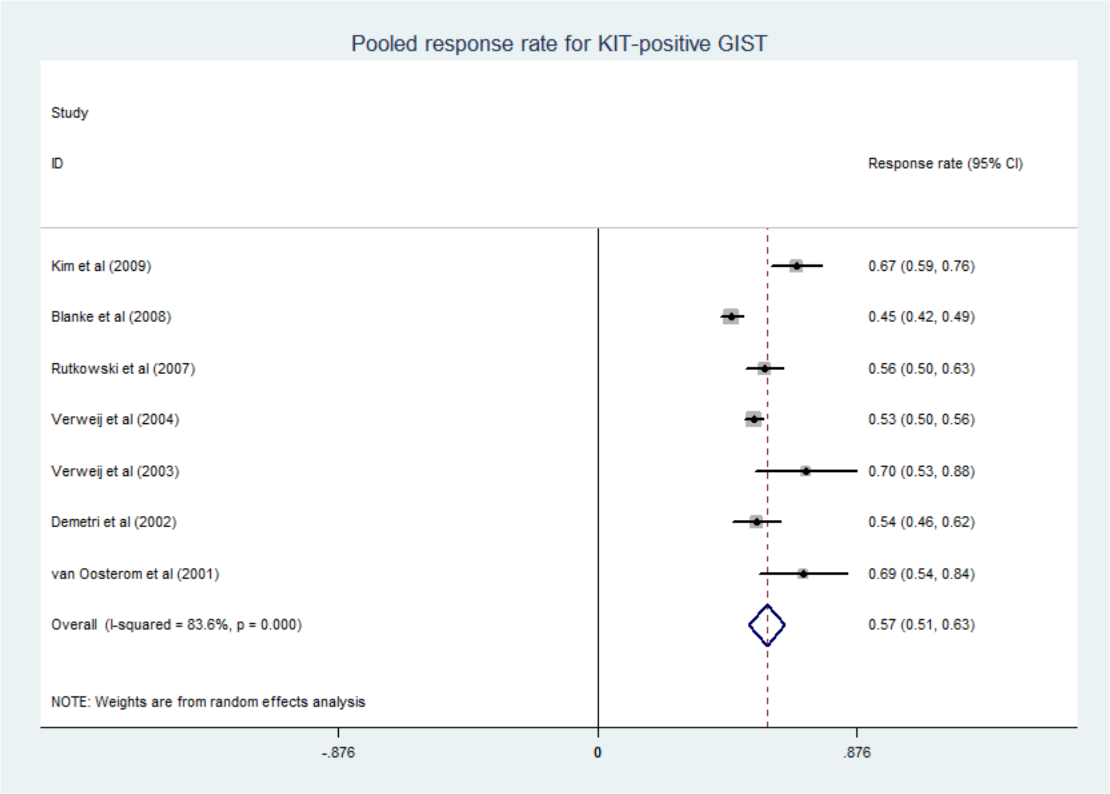

Pooled response rate for KIT-positive GIST
Study
ID
Response rate (95% CI)
Kim et al (2009)
0.67 (0.59, 0.76)
Blanke et al (2008)
0.45 (0.42, 0.49)
Rutkowski et al (2007)
0.56 (0.50, 0.63)
Verweij et al (2004)
0.53 (0.50, 0.56)
Verweij et al (2003)
0.70 (0.53, 0.88)
Demetri et al (2002)
0.54 (0.46, 0.62)
van Oosterom et al (2001)
0.69 (0.54, 0.84)
Overall (I-squared = 83.6%, p = 0.000)
0.57 (0.51, 0.63)
NOTE: Weights are from random effects analysis
-.876
0
.876
