# Supplementary material for: Practical Role of Mutation Analysis for Imatinib Treatment in Patients With Advanced Gastrointestinal Stromal Tumors: A Meta-Analysis
Source: PLoS One. 2013 Nov 4;8(11):e79275. doi: 10.1371/journal.pone.0079275 (PMC3817038; doi:10.1371/journal.pone.0079275)

Funnel plot for response rate of KIT exon 11-mutant GIST vs KIT exon 9-mutant GIST

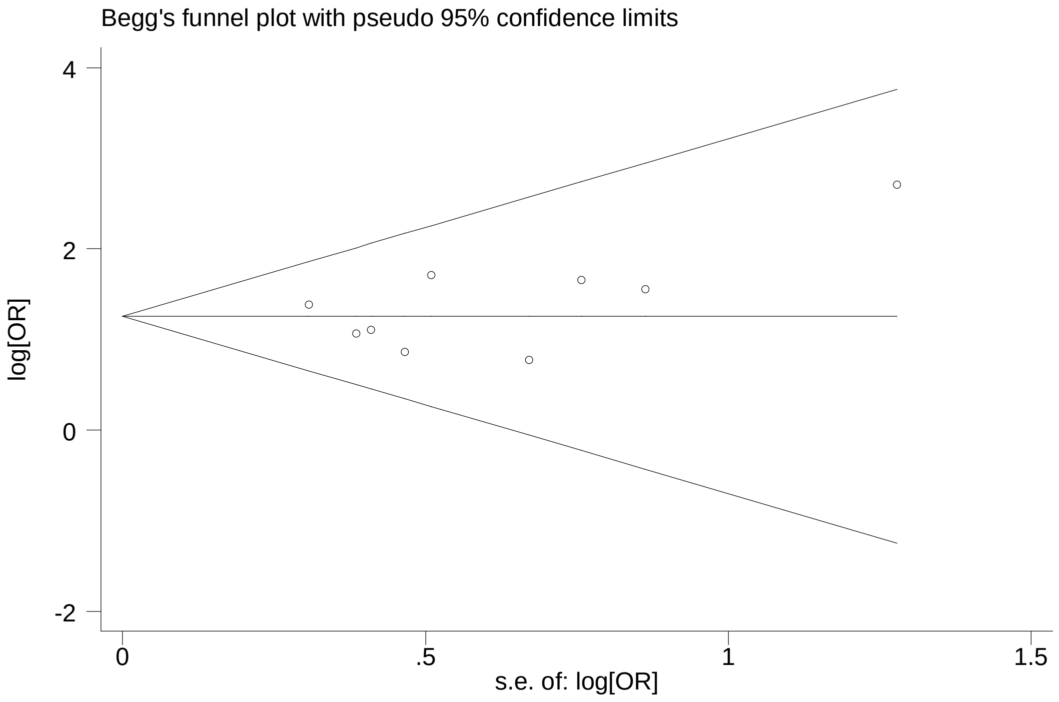


Funnel plot for response rate of KIT exon 11-mutant GIST vs wild type GIST

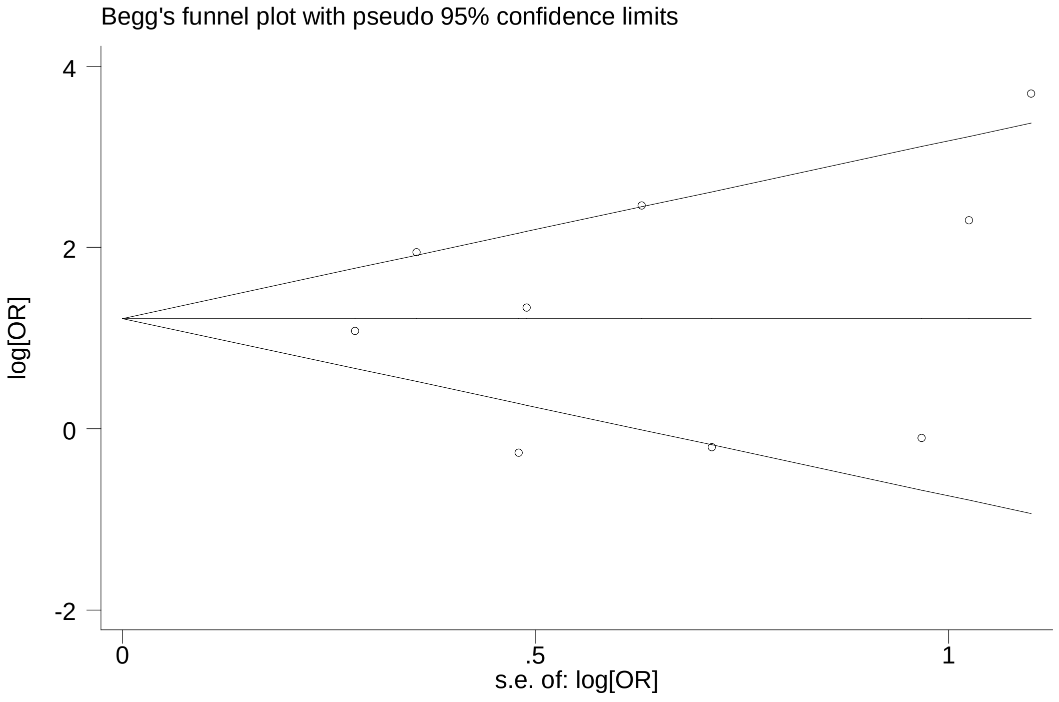

Funnel plot for response rate of KIT exon 9-mutant GIST vs wild type GIST

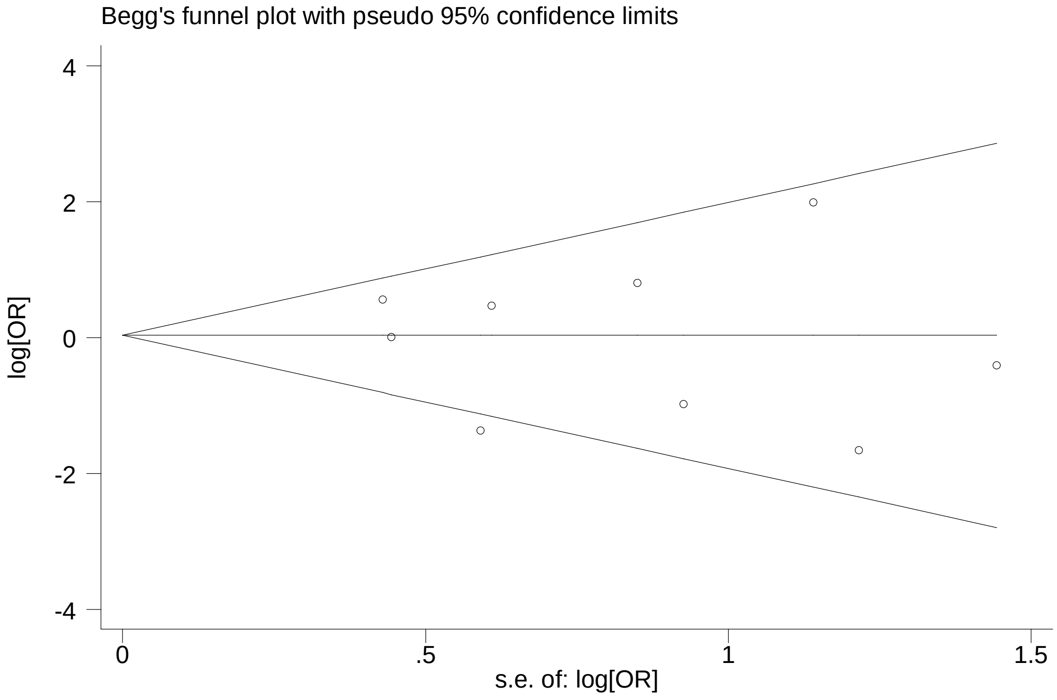


Funnel plot for PFS of KIT exon 11-mutant GIST vs KIT exon 9-mutant GIST

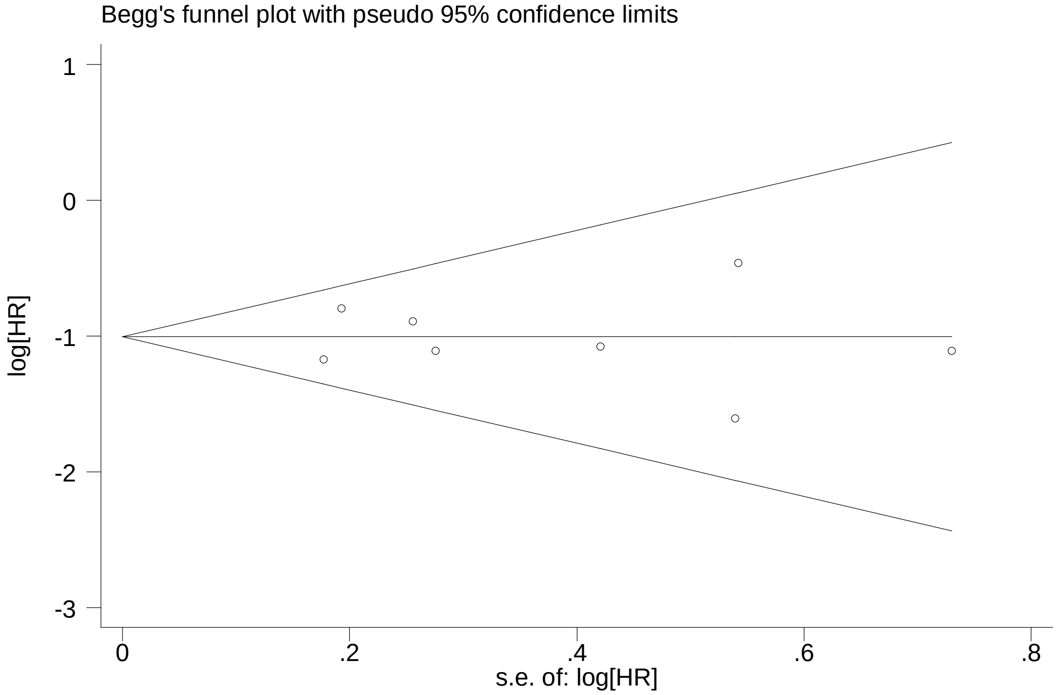

Funnel plot for PFS of KIT exon 11-mutant GIST vs wild type GIST

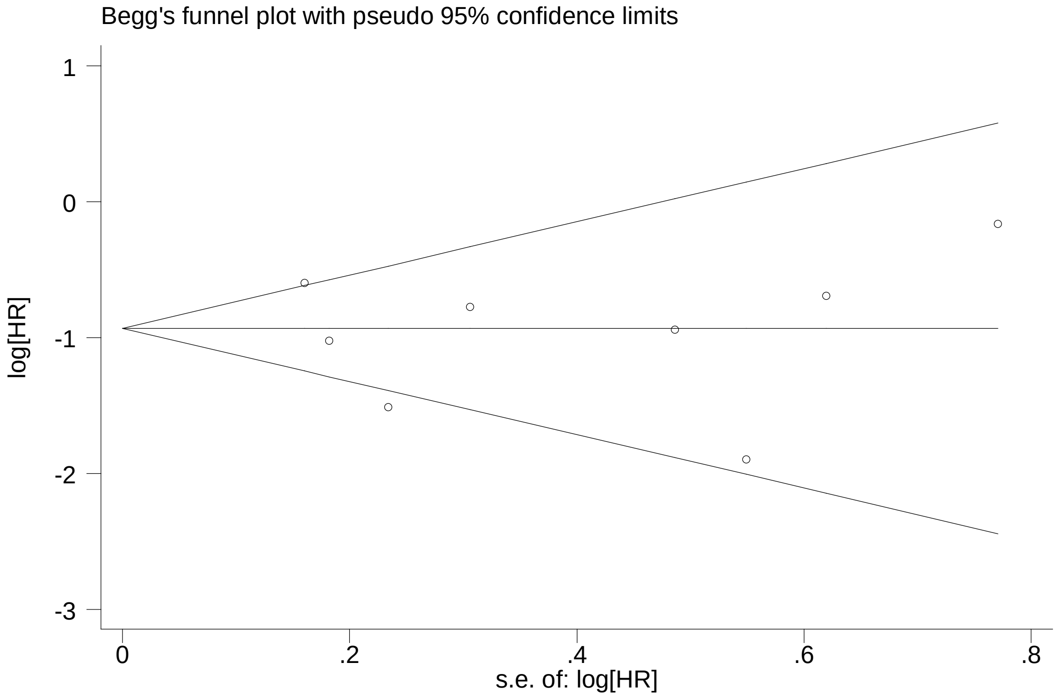


Funnel plot for PFS of KIT exon 9-mutant GIST vs wild type GIST

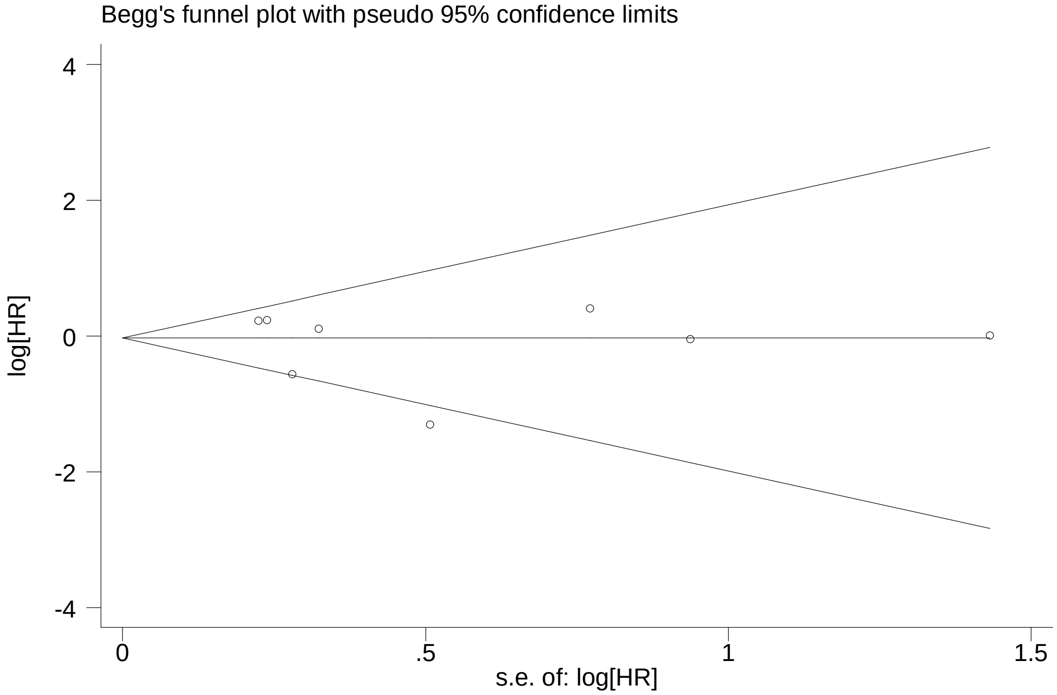

Funnel plot for OS of KIT exon 11-mutant GIST vs KIT exon 9-mutant GIST

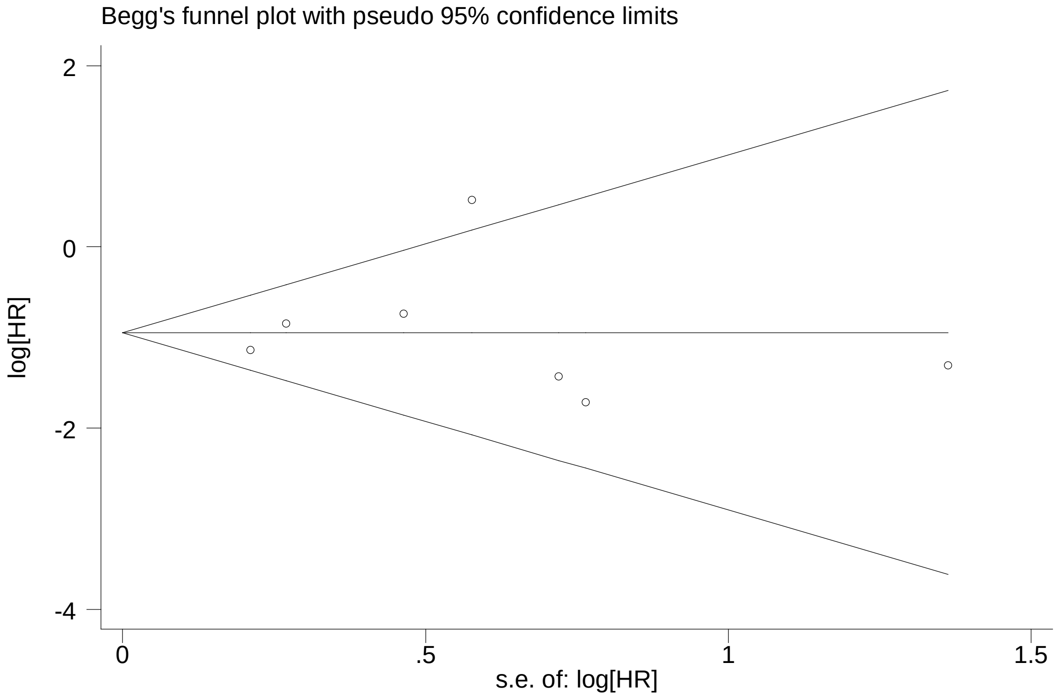


Funnel plot for OS of KIT exon 11-mutant GIST vs wild type GIST

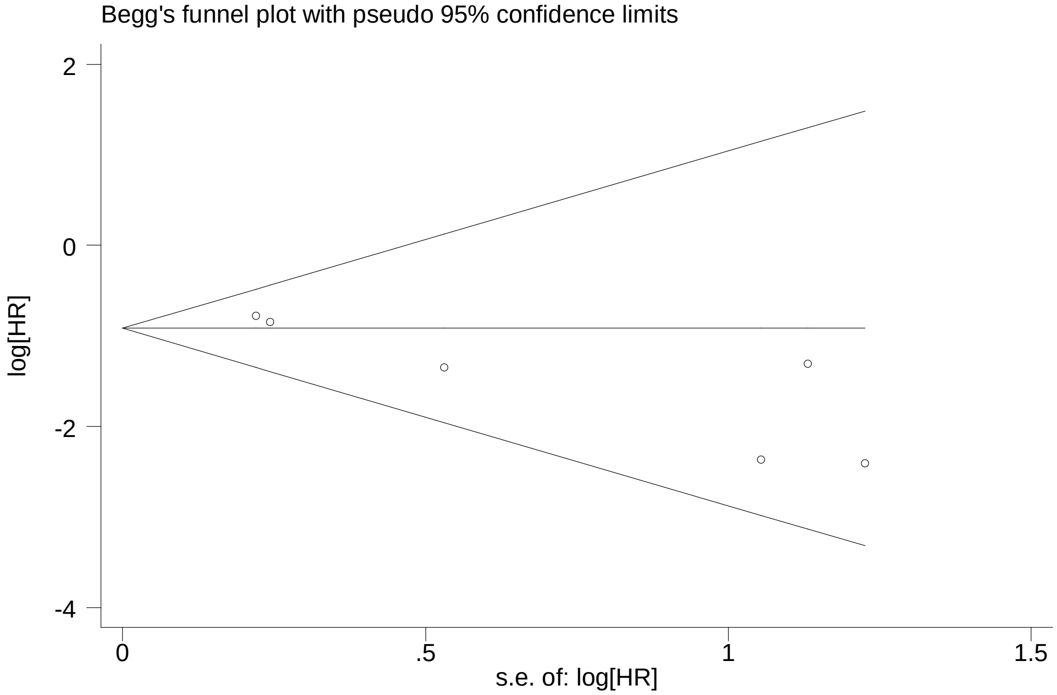

Funnel plot for OS of KIT exon 9-mutant GIST vs wild type GIST

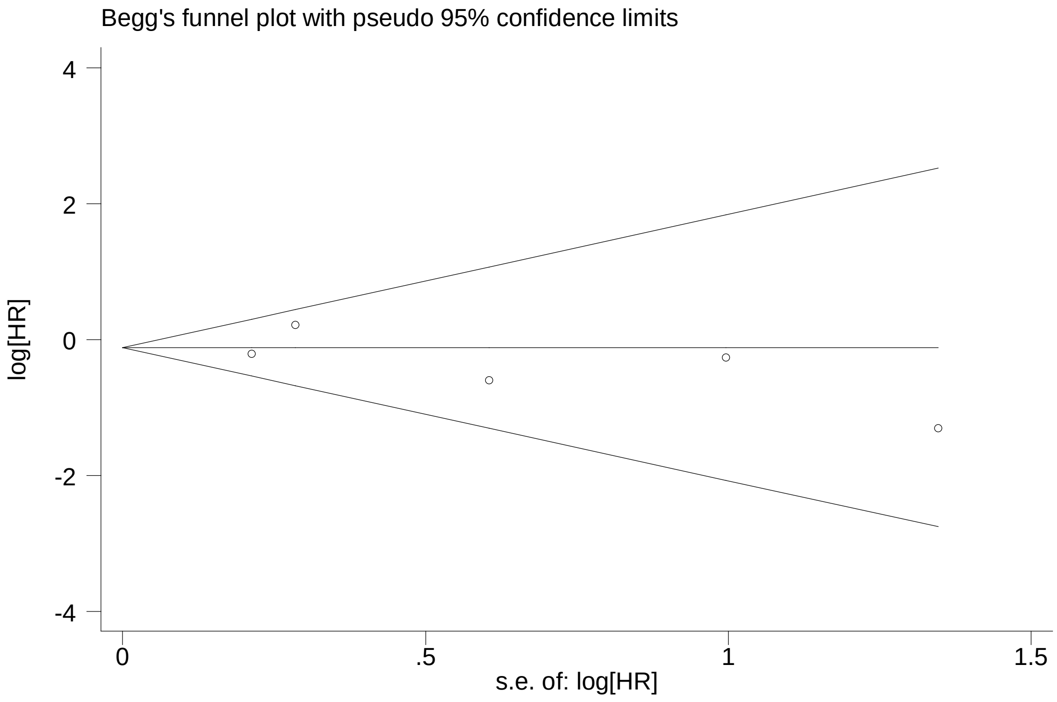


Funnel plot for response rate of KIT exon 11-mutant GIST

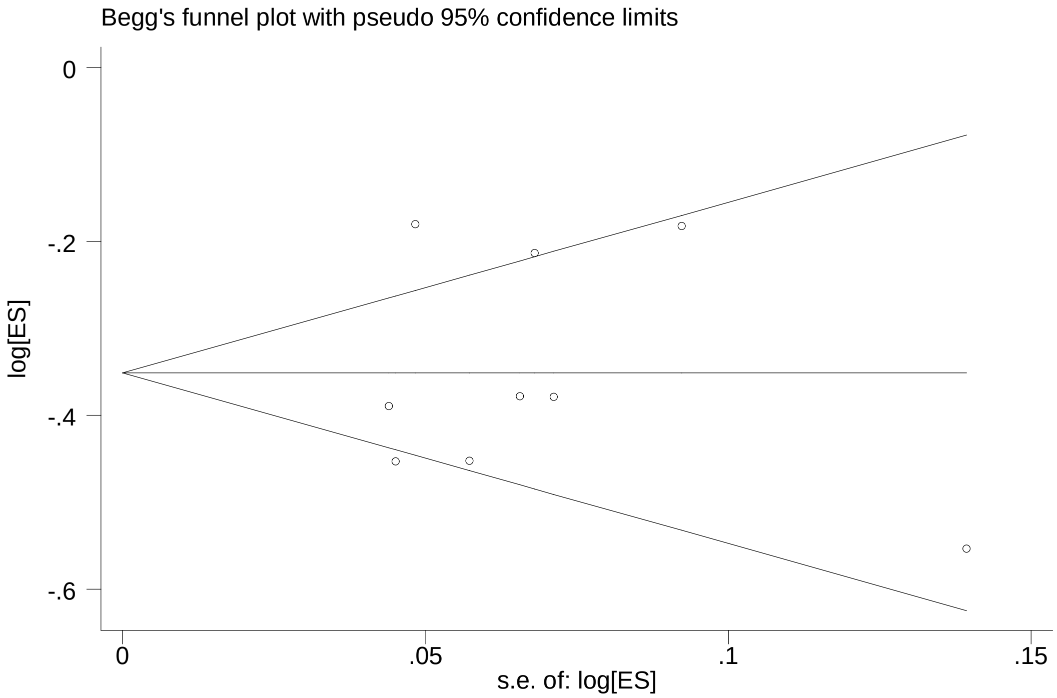

Funnel plot for response rate of KIT-positive GIST

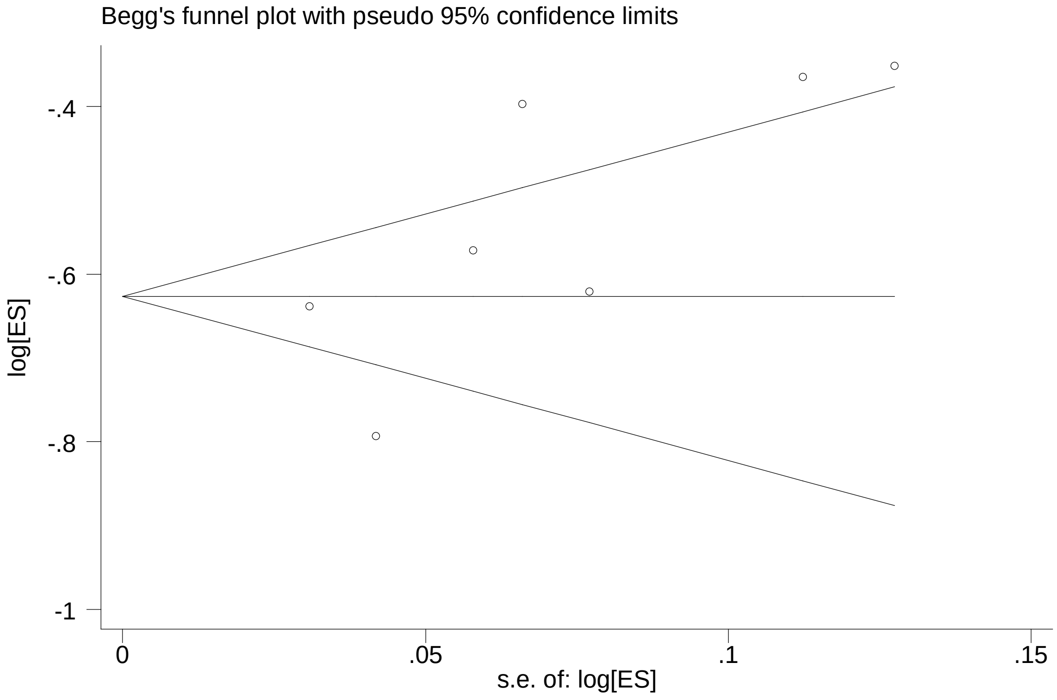

Supplement: Figure S2 — Funnel plots. (PDF) [file pone.0079275.s010.pdf]
